# A combination of annual and nonannual forces drive respiratory disease in the tropics

**DOI:** 10.1136/bmjgh-2023-013054

**Published:** 2023-11-07

**Authors:** Fuhan Yang, Joseph L Servadio, Nguyen Thi Le Thanh, Ha Minh Lam, Marc Choisy, Pham Quang Thai, Tran Thi Nhu Thao, Nguyen Ha Thao Vy, Huynh Thi Phuong, Tran Dang Nguyen, Dong Thi Hoai Tam, Ephraim M Hanks, Ha Vinh, Ottar N Bjornstad, Nguyen Van Vinh Chau, Maciej F Boni

**Affiliations:** 1 Department of Biology and Center for Infectious Disease Dynamics, The Pennsylvania State University, University Park, Pennsylvania, USA; 2 Wellcome Trust Major Overseas Programme, Oxford University Clinical Research Unit, Ho Chi Minh City, Vietnam; 3 Centre for Tropical Medicine and Global Health, Nuffield Department of Medicine, University of Oxford, Oxford, UK; 4 National Institute of Hygiene and Epidemiology, Hanoi, Vietnam; 5 Department of Microbiology, Blavatnik Institute, Harvard Medical School, Boston, Massachusetts, USA; 6 Department of Statistics and Center for Infectious Disease Dynamics, The Pennsylvania State University, University Park, Pennsylvania, USA; 7 Hospital for Tropical Diseases, Ho Chi Minh City, Vietnam

**Keywords:** epidemiology, public health, respiratory infections

## Abstract

**Introduction:**

It is well known that influenza and other respiratory viruses are wintertime-seasonal in temperate regions. However, respiratory disease seasonality in the tropics is less well understood. In this study, we aimed to characterise the seasonality of influenza-like illness (ILI) and influenza virus in Ho Chi Minh City, Vietnam.

**Methods:**

We monitored the daily number of ILI patients in 89 outpatient clinics from January 2010 to December 2019. We collected nasal swabs and tested for influenza from a subset of clinics from May 2012 to December 2019. We used spectral analysis to describe the periodic signals in the system. We evaluated the contribution of these periodic signals to predicting ILI and influenza patterns through lognormal and gamma hurdle models.

**Results:**

During 10 years of community surveillance, 66 799 ILI reports were collected covering 2.9 million patient visits; 2604 nasal swabs were collected, 559 of which were PCR-positive for influenza virus. Both annual and nonannual cycles were detected in the ILI time series, with the annual cycle showing 8.9% lower ILI activity (95% CI 8.8% to 9.0%) from February 24 to May 15. Nonannual cycles had substantial explanatory power for ILI trends (ΔAIC=183) compared with all annual covariates (ΔAIC=263) in lognormal regression. Near-annual signals were observed for PCR-confirmed influenza but were not consistent over time or across influenza (sub)types. The explanatory power of climate factors for ILI and influenza virus trends was weak.

**Conclusion:**

Our study reveals a unique pattern of respiratory disease dynamics in a tropical setting influenced by both annual and nonannual drivers, with influenza dynamics showing near-annual periodicities. Timing of vaccination campaigns and hospital capacity planning may require a complex forecasting approach.

**WHAT IS ALREADY KNOWN ON THIS TOPIC**

- Participatory epidemiology and mHealth studies can generate digital data streams with thousands of data points.
- There is a lack of long-term fine-scale reporting on respiratory infections in the tropics.
- There are diverging views on whether a tropical influenza season exists.

**WHAT THIS STUDY ADDS**

- This study provides a 10-year daily time series on influenza-like illness (ILI) from a community-led mHealth study.
- Both annual and nonannual cycles are identified as influential, with climate factors showing weak associations with influenza and influenza-like illness patterns.
- The inferred annual cycle manifests as a short low incidence season rather than a short high incidence season.

**HOW THIS STUDY MIGHT AFFECT RESEARCH, PRACTICE OR POLICY**

- The existence of a nonannual cycle driving respiratory disease dynamics suggests that forecasting the next ILI peak may be possible.
- The ability to forecast high respiratory disease incidence may help with vaccination planning and hospital preparedness.

## Introduction

The seasonality of respiratory diseases in tropical regions is less well studied than in temperate regions. One of the challenges is the limited scope of surveillance systems in tropical regions as healthcare resources are prioritised to higher burden diseases such as malaria, tuberculosis, HIV and dengue. Temperate regions, in contrast, have long-term established surveillance systems for respiratory disease as this is the highest priority infectious disease burden in most temperate countries. Thus, the burden of respiratory disease in the tropics may be underestimated because of the paucity of data.^1 2^ Some recent studies^3–8^ have shown that respiratory diseases are associated with morbidity and mortality in tropical regions, but more data are needed for policy-making.

Another difficulty of studying respiratory disease dynamics in the tropical regions is that irregular epidemic patterns are typically observed. The absence of winter in tropical regions makes the yearly patterns of influenza and influenza-like illness (ILI) less seasonal and less predictable.^9^ Thus far, it has been found that influenza in tropical regions shows lower variation in incidence,^10 11^ higher variation in epidemic timing^10 12^ and different periodicities geographically,^10 13–18^ making it difficult to forecast periods of high incidence. Although some studies in tropical areas have shown associations between climate or environmental factors and influenza transmission,^11 19–23^ this relationship remains elusive^16 24 25^ and caution is needed in interpreting these results and drawing inferences on a ‘typical’ tropical influenza season.

There are two common shortcomings in many past analyses focused on influenza and ILI seasonality in tropical areas. First, it is not possible to generate robust evidence for seasonality using short time series with monthly data.^26–30^ Short time series may be unrepresentative of longer-term behaviours, and when combined with a monthly stratification of cases, provide low statistical power to determine when the respiratory disease season occurs if there is one. This may be caused by the lack of data given the limited surveillance system in tropical regions. Second, it is not sufficient to base an analysis on associations between climate factors and ILI/influenza incidence when describing seasonality, as has been done previously,^21 22 31 32^ because spurious associations will be common between an annually structured set of climate factors and an event like an ILI epidemic that occurs historically about once per year. A determination of whether seasonality exists is needed first. Quantitative descriptions of long-term fine-scale time series are needed to accurately characterise the presence and pattern of seasonality in respiratory disease incidence.

Here, we present the periodic signals detected from 10 years of daily influenza-like-illness (ILI) reports and 7.5 years of molecular surveillance for influenza virus, collected from a community mHealth syndromic surveillance study in Ho Chi Minh City (HCMC), Vietnam. Using time series decomposition and regression models, we aimed to identify periodicities in the ILI and influenza time series, and to evaluate the explanatory power of these periodicities on both high-incidence and low-incidence periods of ILI and influenza.

## Methods

### Literature review

We conducted a literature review to determine the value of our study in this field. We searched PubMed for studies matching the search terms “((influenza[Title/Abstract] OR “respiratory disease”[Title/Abstract]) AND (seasonality[Title/Abstract])) AND (tropic*[Title/Abstract])”, identified 127 studies spanning 2003–2023 (excluding our mid-study analysis),^33^ and evaluated them on four criteria: (1) statistical tests for the presence or absence of seasonality, (2) weekly/daily reporting of respiratory disease incidence counts or percentages, (3) a minimum of 5 years of data collection and (4) a minimum of one thousand or ten thousand incidence data points available per location. Nineteen studies were reviews (excluded), and another thirty were excluded as they were laboratory studies, phylogenetic analyses, models or analyses for temperate or sometimes combined temperate-subtropical zones. Of the remaining 78 studies, 65 had no statistical tests for presence or absence of seasonality. Some studies used a visual signal or an aggregation of data (across years) to the month/week level to describe a season. Many studies reported associations with climate variables, an analysis that does not establish whether a disease incidence pattern is annual or nonannual. Seven of the remaining 13 studies used monthly data (too coarse for seasonality analysis) or had fewer than 5 years of data (insufficient statistical power to identify a season). Six studies^13 15 17 34–36^ met criteria (1) through (3) with sample sizes of number of incidence counts/percentages equal to N=520, 520, 988, 3640, 6916 and 9828. The largest of these, a Brazilian study^17^ showing a lack of seasonality in tropical but not subtropical parts of Brazil, is the largest and most comprehensive study to date on the presence/absence of respiratory seasonality in the tropics. The other two studies with >1000 data points presented seasonality analyses for subtropical China.^15 34^

### Study design

Starting in August 2009, the Oxford University Clinical Research Unit (OUCRU) in Ho Chi Minh City (HCMC), Vietnam began recruiting community outpatient clinics to participate in a daily ILI reporting programme by standard mobile phone short messaging services (SMS). A total of 89 clinics were recruited during the first 5 years of the study, and data collection ended on December 31, 2019. The recruitment process began by contacting physicians at the Hospital for Tropical Diseases in HCMC who also ran their own private clinics, and recruitment proceeded by word of mouth from the initial participants, through annual community engagement meetings, and by canvassing local communities and distributing leaflets and other information on the project. We do not know the refusal rate for participation. The study was run in collaboration with local clinicians, community leaders, and institutions, as described in the reflexivity statement (online supplemental file 2). Clinicians or nurses in each participating clinic sent daily text messages to OUCRU reporting the total number of visits, the number of patients that had ILI symptoms, and the number of hours that the clinic was open that day. The ECDC ILI definition was used: (1) sudden onset of symptoms within the past 3 or 4 days; (2) one or more of the following general symptoms (a) fever with axillary temperature above 37.5°C, (b) malaise, (c) headache or (d) myalgia and (3) one or more of the following respiratory symptoms (a) cough, (b) sore throat or (c) shortness of breath.^37^ Three common ILI definitions exist with minor differences in sensitivity and specificity.^38–40^

10.1136/bmjgh-2023-013054.supp2 Supplementary data

The percentage of ILI patients among total outpatient visits per day (%ILI) in each clinic from 1 January 2010 to 31 December 2019 is used as the primary data type in the analysis.^41^ The ILI ζ-score is defined as the ratio of the %ILI observed on a single day divided by the mean %ILI in a 365-day moving window (see online supplemental text S1) to remove long-term decreasing ILI trends seen in seven clinics.^12 33^ Daily data were detrended using the ζ-score for each clinic, and then the arithmetic mean of ζ-scores among all clinics reporting that day was calculated to obtain an aggregated ILI time series. This aggregated all-clinic ζ-score was smoothed with a 7-day moving average to remove weekend effects.

10.1136/bmjgh-2023-013054.supp1 Supplementary data

Starting 23 May 2012, 24 of the participating outpatient clinics agreed to participate in additional molecular influenza surveillance. Based on a randomised schedule, one or two clinics per week were assigned to collect nasopharyngeal swab samples for influenza molecular confirmation by reverse transcription PCR (RT-PCR).^33^ Samples were subtyped to identify A/H1, A/H3 and influenza B. Counts of molecular samples and the number testing positive for each subtype were aggregated into 21-day windows to ensure a sufficient sample size in each window. A daily ILI+ time series^41 42^ was constructed as the product of the influenza positivity rate each day (this is constant for 21-day stretches) and the aggregate all-clinic daily ILI ζ-score. The daily ILI+was then smoothed with a 7-day moving average.

### Statistical analysis

We used autocorrelation functions, discrete Fourier transforms and wavelet analyses to identify periodic signals in the ILI ζ-score and ILI+ time series. We then used simple cyclic step functions (called ‘cycles’ in equations below) to infer the magnitude and timing of periodic fluctuation in the time series (see online supplemental text S3).

To identify the predictive ability of the inferred cycles in the ILI data, we regressed the ILI ζ-score using a lognormal model on a range of potential predictors including the inferred cycles, 7-day lagged ILI ζ-score, 12 climate covariates and a school-term indicator (equation 1).

(1) $$\begin{array}{ll} \ln\left(E\left(\zeta_{i}\right)\right)= & \beta_{0}+\beta_{1}\zeta_{i-7}+\beta_{2}school_{i}+\\ & \beta_{3-4}cycles_{i}+\beta_{5-16}climate_{i} \end{array}$$

The 7-day autoregressive term was included because human-transmissible pathogen incidence time series are temporally autocorrelated. Climate data were collected from the NASA POWER Project^43^ and 12 climate covariates were included: temperature, absolute humidity and rainfall, all lagged at 0, 1, 2 and 3 weeks, as all have been reported to be associated with ILI or influenza trends in previous studies.^11 44 45^ The climate covariates were scaled using z-score normalization. School term was included because of the high transmissibility of ILI among children (details in online supplemental text S4).^46 47^

In the molecular influenza time series, the statistical approach needs to account for an over-representation of zeroes in the ILI+ time series (about 9% of daily time points). We use a two-step gamma hurdle model to regress ILI+ onto covariates. The first step is a logistic model estimating the probability that influenza activity is present given the predictors (equation 2). The second step is a gamma model estimating the magnitude of influenza activity conditioned on influenza activity being present on that day (equation 3).

$\begin{array}{ll} step1:logit\left(P\left(ILI_{i}^{+}>0\right)\right)= & \beta_{0}+\beta_{1}ILI_{i-21}^{+}+\beta_{2}school_{i}+\\ & \beta_{3-4}cycles_{i}+\beta_{5-16}climate_{i} \end{array}$(2)

(3) $$\begin{array}{ll} step2:\ln\left(E\left(ILI_{i}^{+}|ILI_{i}^{+}>0\right)\right)= & \beta_{0}+\beta_{1}ILI_{i-21}^{+}+\beta_{2}school_{i}+\\ & \beta_{3-4}cycles_{i}+\beta_{5-16}climate_{i} \end{array}$$

The autoregressive term of ILI+ is 21-day lagged ILI+. The inferred cycles were estimated from ILI+ data. All the other predictors remain the same as in Eq .1.

To compare results in HCMC to locations with known seasonality, we collected ILI data from temperate regions, including US and four European countries (details in online supplemental text S2). For both HCMC and US ILI data, we conducted stepwise AIC (Akaike Information Criterion)-based forward model selection to select the predictors that contribute substantially to the goodness-of-fit of the model, measured as R^2^. Specifically, we allocated the R^2^ to each predictor by calculating how much explained variance will increase when adding the predictor into the model (details in dominance analysis^48–50^ and online supplemental text S5). In this way, we are able to compare the contributions (in terms of explained variance) of the annual and nonannual cycles to ILI trends in HCMC and the US.

All analyses were conducted using R version 4.0.3. Wavelet analysis was done using WaveletComp package.^51^ Gamma hurdle models were implemented using glmmTMB package.^52^ R^2^ decomposition was done using relaimpo package.^53^

## Results

From 1 January 2010 to 31 December 2019, 89 clinics were enrolled in the study. A total of 66 799 SMS text messages with ILI reports were sent covering 2 893 515 outpatient visits, 257 789 (8.9%) of which were patients meeting the clinical definition of ILI. Among the clinics, 33 were selected for analysis as they sent more than 300 reports during the 10-year period with >50% of reports showing a non-zero number of ILI patients. The selected clinics were evenly distributed across neighbourhoods of HCMC (online supplemental figure S1). To evaluate biases in individual physician diagnosis of ILI, we compared ILI reporting rates to confirmed influenza trends and did not find patterns of clinics consistently overdiagnosing or underdiagnosing ILI relative to concurrent influenza circulation (online supplemental figure S2). Among the included clinics, the median daily number of patients per clinic was 44 (IQR: 35–53), and the median of the daily number of patients per clinic meeting the definition of ILI was 4 (IQR: 3–6).

### Periodic signals in syndromic influenza-like illness data

**Figure 1:**
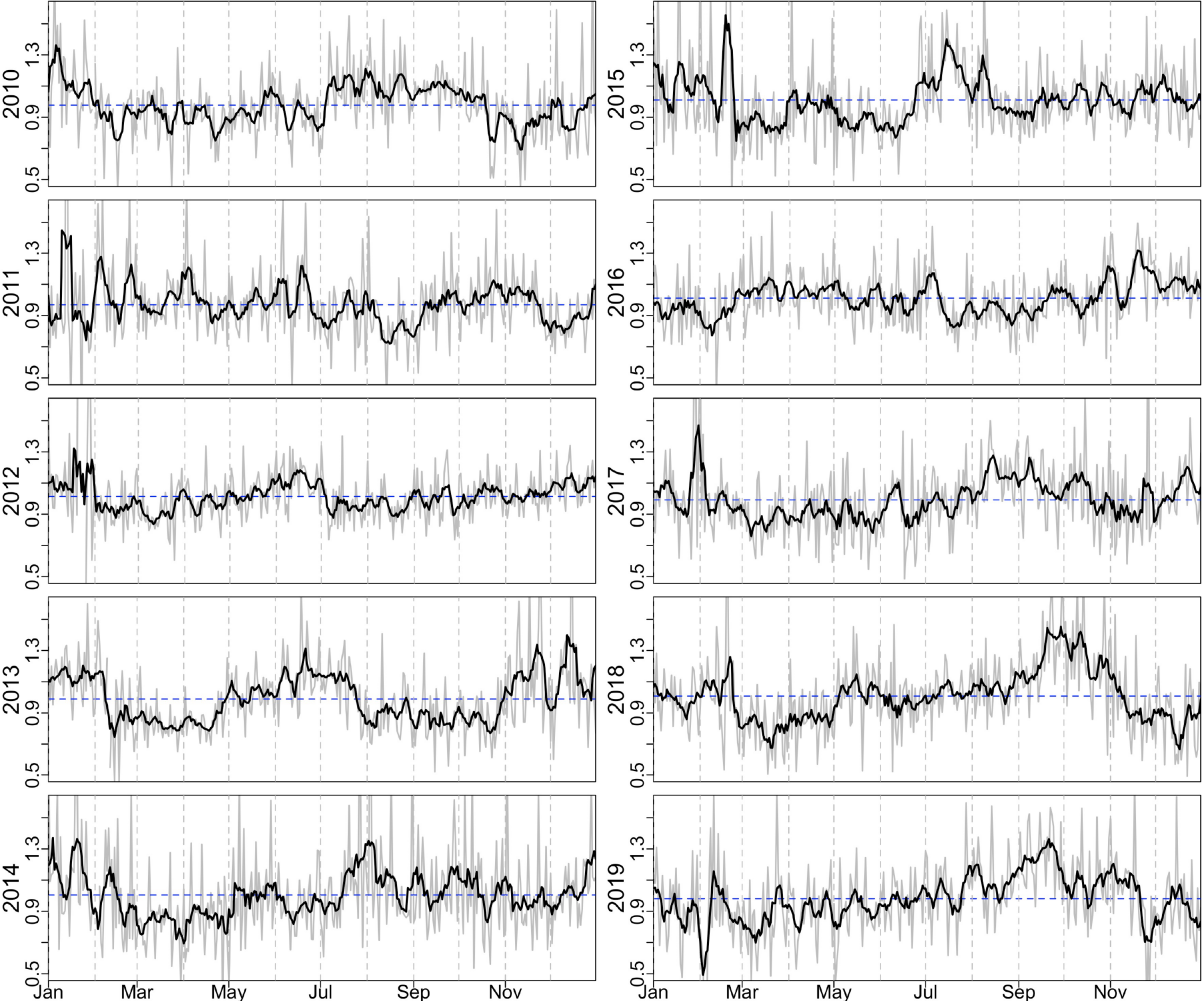
Daily ILI ζ- score (grey line) and 7-day smoothed ILI ζ- score (black line) from 2010-01-01 to 2019-12-31. The mean of ILI ζ- score in each year is shown as a blue horizontal dashed line.

The syndromic ILI ζ-score time series appears noisy (but is not white noise, p<0.001, Box-Ljung test) and exhibits weak fluctuations with no visually discernible seasonality (figure 1), especially when compared with ILI patterns in temperate regions (online supplemental figure S3). The absence of strong and regular seasonality is consistent with subtropical Hong Kong and tropical Singapore (online supplemental figure S3). As the seasonal signals are not visually obvious, three separate analyses were used to determine the presence/absence of periodic signals in the data.

**Figure 2:**
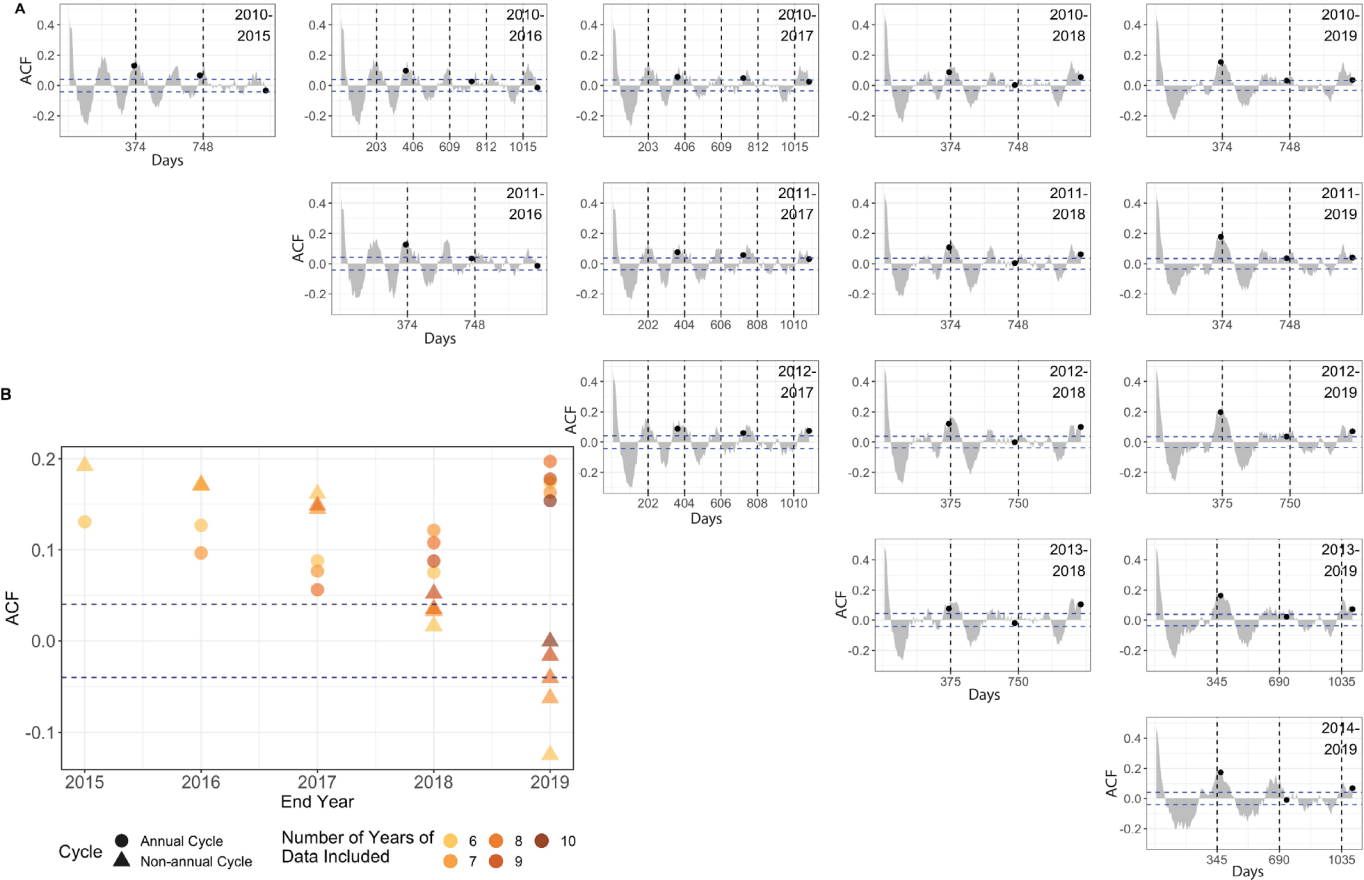
Nonannual and annual cycles in ILI ζ- score. (A) Pearson autocorrelation function (ACF) of ILI ζ- score timeseries, split across different study periods. Horizontal dashed lines label the regions where ACF is significantly different from 0 (p < 0.05). Vertical dashed lines label the peak lag period of ACF between 150 to 450 days. Annual cycle is labeled with a black dot. Periods are inclusive so “2010-2015” spans six years. (B) The shift of ACF values from nonannual cycles to annual cycles. The x-axis denotes the last year included in the time series, and the y-axis shows the ACF value. ACF values of annual cycles (circles) and nonannual cycles (triangles) are shown. The nonannual cycle showed stronger signals prior to 2017.

Periodic signals detected by autocorrelation function (ACF) were weak in HCMC compared with temperate regions (online supplemental figure S4), and they were not robust to the number of years included in the data. As in our previous analysis,^33^ the first 8 years of data collection showed a well-supported 203-day signal from 2010 to 2017 (figure 2A, top-left panels) and an annual signal appearing for most time periods. However, including all 10 years of data from 2010 to 2019 showed a strong annual signal without a nonannual signal; this appears to be driven primarily by the high inter-year correlation in the ILI signals between 2017/2018 and 2018/2019 (Pearson’s *ρ*=0.325 and 0.568, respectively, online supplemental figure S5). This shift from a primarily nonannual cycle to primarily annual cycle (figure 2B) is also observed in the wavelet analysis (online supplemental figure S6). Statistical evidence (via the ACF) for both annual and nonannual cycles is robust to sub-setting the time series to shorter periods (figure 2B), except for the year 2019 which appears to have a singularly strong effect on the autocorrelation patterns. Discrete Fourier transform of the ILI ζ-score supports both a nonannual cycle (215 days) and an annual cycle (365 days) showing equally strong signals (online supplemental figure S7).

**Figure 3:**
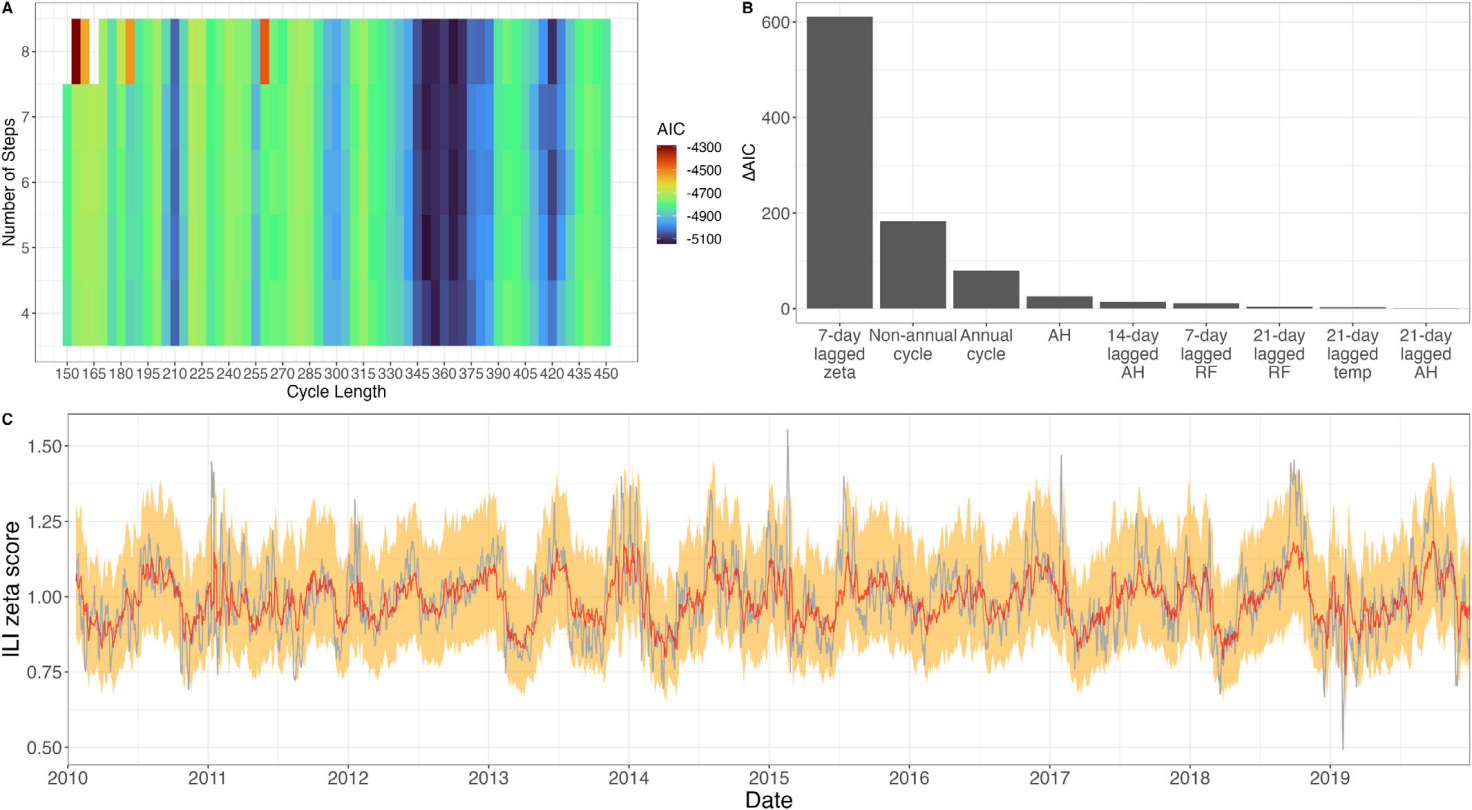
Nonannual and annual cycles in ILI ζ- score. (A) The AIC of cyclic step functions for ILI ζ-score for different numbers of steps and different cycle lengths. AIC is lowest for a cycle of 210 days or 365 days. (B) Contribution of each predictor is calculated as the AIC difference when removing the predictor from the fitted lognormal model. “Temp” denotes temperature, “RF” denotes rainfall, and “AH” denotes absolute humidity. (C) Predicted values of ILI ζ-score (red) and 95% prediction intervals (orange) of the full model including both cycles; the observed ILI ζ-score is shown in grey.

To describe the cycles quantitatively, a fit of a cyclic two-step function to the ILI ζ-score selected 365 days and 210 days as the two periodicities most likely to explain the data (AIC=−5016 and AIC=−4931, respectively). For the annual cycle, the ILI ζ-score is 8.9% (95% CI 8.8% to 9.0%) lower from 24 February (95% CI 24 February to 25 February) to 15 May (95% CI 12 May to 18 May), suggesting that respiratory disease seasonality in the tropics may manifest itself as a low season rather than a high season. For the 210-day cycle, the ILI ζ-score is 6.8% (95% CI 6.6% to 7.0%) lower for a 104-day period of the cycle. In both cases, the difference in respiratory disease incidence between low and high season is small. An 8-step 365-day cycle (AIC=−5138) and a 5-step 210-day cycle (AIC=−5033) were selected from a varying number of steps and cycles (figure 3A, online supplemental figure S8) and were included in the regression analysis. After stepwise AIC-based model selection, annual and nonannual cycles are retained in the final regression along with the 7-day autoregressive term and various climate factors mainly related to humidity (figure 3C) (online supplemental table S1). The AIC difference when removing the nonannual cycle (ΔAIC=183) from the lognormal model was larger than when the annual cycle was removed (ΔAIC=79), indicating that nonannual trends contain specific information for the ILI incidence pattern that is not contained in other predictors. The large contribution of the nonannual cycle to the model’s goodness-of-fit may signal the presence of certain nonannual epidemiological processes unique to tropical regions. The larger AIC difference when removing all annual covariates (ΔAIC=263) suggests that ILI incidence showed a stronger annual pattern (data-derived and not necessarily climate-linked). The ΔAIC for all the climate factors alone is 50.

Critically, the nonannual ILI periodicities observed in HCMC are not present in temperate datasets that were processed with the same methods used for the HCMC data. Autocorrelation functions (online supplemental figure S4) and wavelet analysis (online supplemental figure S6) show strong peaks at 1 year with no signs of subannual periodicity—this is consistent across regional US data sets (10 HHS regions) and 4 European countries with time series longer than 5 years. Based on an R^2^ decomposition^48^ from the regression models from HCMC and 10 HHS regions, the nonannual inferred cycle explains around 15% variance of the ILI ζ-score in the tropics compared with <1% of the variance in 10 HHS regions (online supplemental figure S9). Weaker annual signals were observed in HCMC than in temperate zones, but the nonannual signals were stronger.

### Periodic signals in influenza data

In the molecular surveillance component, a total of 2604 nasal swabs were collected from 23 May 2012 to 31 December 2019 of which 21.2% (N=559) were positive for influenza. After subtyping, 6.3% were positive for influenza H1N1, 6.5% were positive for influenza H3N2, and 8.0% were positive for influenza B. There was no significant correlation between syndromic and virological data (Pearson’s ρ=−0.14, p=0.11), suggesting cocirculation of many non-influenza respiratory pathogens. Between 23 May 2012 and 23 January 2019, of 2225 samples collected, 1156 (52.0%) were from male patients and 1069 (48.0%) from female patients.

To validate the molecular surveillance trends in our community-based study, we compared our pattern of ILI+ to the ILI+ time series seen in hospital-based surveillance via Vietnam’s National Influenza Sentinel Surveillance System in HCMC.^12 54–56^ The overlap between 2012 and 2015 for a period of 186 weeks between the two time series showed similar circulation pattern of influenza and its subtypes (Pearson’s correlation ρ=0.568 (95% CI 0.456 to 0.662) for overall influenza, ρ=0.784 (95% CI 0.719 to 0.836) for subtype A/H1N1, ρ=0.706 (95% CI 0.621 to 0.774) for subtype A/H3N2 and ρ=0.523 (95% CI 0.404 to 0.624) for influenza B; all p<10^−4^, online supplemental figure S10).

**Figure 4:**
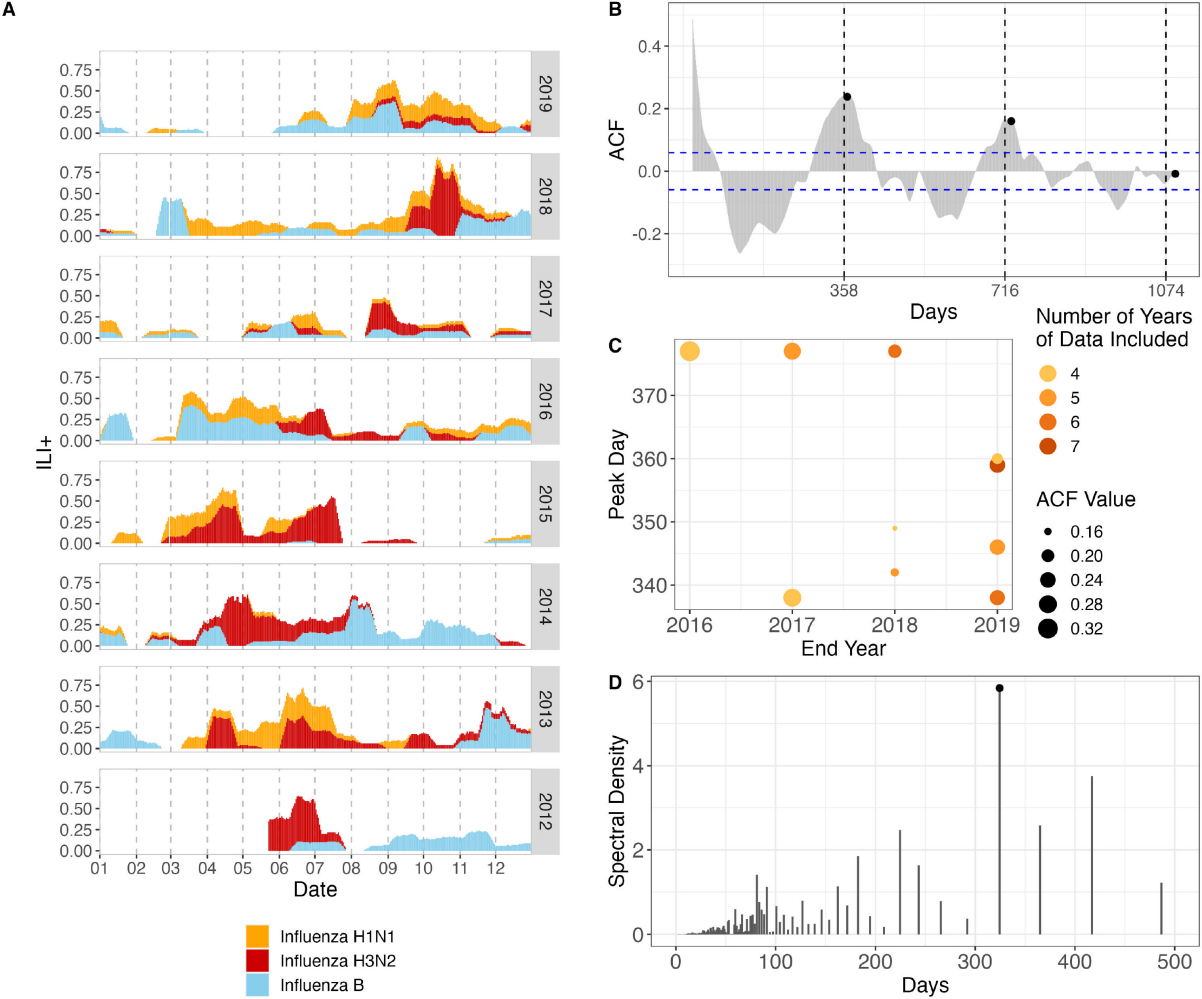
ILI+ time series and periodic signals. (A) The 7-day smoothed overall ILI+ stacked by subtypes. (B) ACF plot of the entire time series of total ILI+. Vertical dashed line labels show ACF peaks at 358-day lag and the subsequent cycles. Black points show annual cycles. (C) The peak lag of ACF varied between 338 and 377 days when varying the length of the included ILI+ time series. (D) Discrete Fourier transform of the entire time series of overall ILI+. The black circle labels the dominant 324-day cycle.

**Figure 5:**
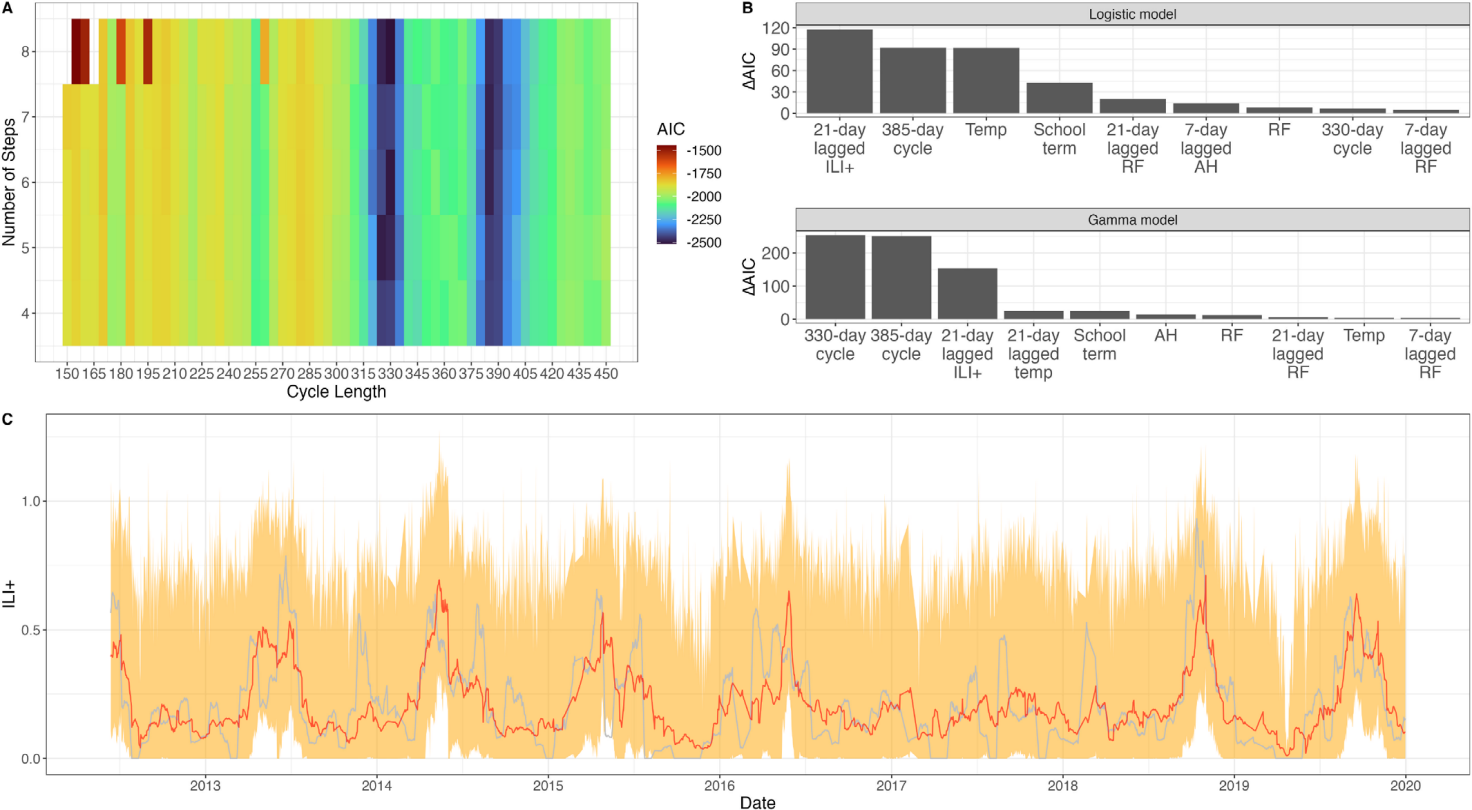
Cycles in ILI+. (A) AIC heatmap for cycle length and the number of steps allowed in each cycle. Near-annual cycle lengths (330-day and 385-day) have the best fits. (B) AIC contribution of each predictor in a gamma hurdle model, measured as the AIC difference when removing the predictor from the final model. “Temp” denotes temperature, “RF” denotes rainfall, and “AH” denotes absolute humidity. (C) The predicted ILI+ (red) with the 95% prediction interval (orange) is shown with the observed ILI+ (grey).

There is no conspicuous seasonality in influenza incidence patterns in HCMC (figure 4A). There appears to be a single influenza peak per year, with autocorrelation peaking at 358 days (figure 4B) and showing peak values around (but not on) the annual cycle when changing the number of years of data included (figure 4C). Discrete Fourier transform showed the highest power at the 324-day cycle (figure 4D), and the cyclic step functions showed the highest likelihood at periods of 330 days and 385 days (figure 5A, online supplemental figure S8), indicating near-annual periodicities in the influenza pattern in HCMC. As in previous analyses, a single influenza peak per year does not guarantee that the timing is repeatable or consistent.^12^ In addition, the inconsistent timings and peaks across subtypes (online supplemental figure S11) suggest a lack of climate or school-term influence on influenza circulation. The best-fit gamma hurdle model explaining the ILI+ data included both the 330-day and 385-day cycles (online supplemental table S2, figure 5C). The multiple near-annual periodicities in ILI+ suggest a period with high influenza activity that keeps shifting every year, a hypothesis that would require further testing.

## Discussion

Our community-led syndromic mHealth study was designed to remove barriers to enrollment, simplify reporting and encourage long-term consistent participation in order to generate a syndromic data stream comparable to the ‘big data’ epidemiological outputs that began to be assembled at the beginning of last decade. With the integration of digital data streams into epidemiological analysis,^57–61^ data streams with N=10^8^ data points can be easily assembled and mined for associations with disease incidence data. As with any data set of this size, some of these associations are spurious.^62^ Our purpose here was to generate a medium-sized data stream of ~10^5^ data points (here >250 000 patients meeting the ILI case definition) where each data point was traceable back to a physician’s diagnosis of a patient’s symptoms presentation. The proof of principle that a ‘medium data’ approach can work at this scale is the validation of our community study’s influenza time series against Vietnam’s national sentinel surveillance system showing the same incidence patterns for influenza A/H3N2, A/H1N1 and influenza B over a 186-week period. Additionally, the daily reporting in this study provides a unique level of resolution in identifying annual or cyclic patterns of disease incidence.

In previous studies aiming to characterise seasonality of influenza in tropical regions, it has been common to assume the existence of seasonal trends. Seasonality in tropical locations has been described in previous research by identifying that epidemics occur each year, without seeking to determine whether there is consistency in their timing.^10 19 22 26 63–65^ Rather than noting whether each calendar year contains an influenza epidemic in HCMC, we incorporated inferred cycles into our statistical models that estimate best-fitting cycle lengths. These inferred cycles, and their predictive ability in the regression models, provide evidence of the existence of cycles with lengths different from 365 days. An identical approach to temperate ILI data does not show the existence of such cycles.

### Mechanisms

There is unlikely to be a specific climate effect driving ILI or influenza dynamics in the tropics. In our analysis, climate covariates tend to have low explanatory power (figures 3B and 5B). Nevertheless, the inferred low ILI season (24 February to 15 May) does occur during the hottest and driest months in HCMC, suggesting this link deserves further investigation despite the mixed associations between climate and ILI seen in online supplemental table S1. Different influenza subtypes peak at different times of year (figure 4A), leading to a conclusion of an improbable link between climate factors and the particular influenza subtype they are influencing.

Seasonality explanations in temperate regions are clearer: the environmental factors associated with viral survival and transmissibility and human movement/contact behaviours all experience abrupt changes during winter. With no winter forcing in the tropics, we may be observing the disease dynamics and their associated cycles driven by other immune, environmental or demographic factors. That is, the observed periodic signals in our study may be indicative of the natural internal clockwork of the dynamics of respiratory viruses when winter is absent. Another possibility is that the dynamics are fluctuating around an endemic equilibrium under the effect of stochasticity (eg, super-spreading events, importation) when the environmental drivers are weak, leading to transient dynamics, with possibly cyclic behaviours.^66–68^

Second, a lack of correlation between ILI incidence and PCR-confirmed influenza incidence indicates that a broader understanding of all respiratory virus circulation will be necessary to understand what is perhaps a more complex system with many viruses competing for resources, excluding other viruses through short-term immunity mechanisms, and driving a pattern of incidence in humans that is visible to us as a series of low peaks and long shallow troughs of ILI.^69^ A long-term cohort with a weekly panel of molecular diagnostics would be a beneficial starting point for describing the interactions among a large group of respiratory viruses.

Finally, a reassessment of the definitions of ILI or influenza season is needed in characterising respiratory virus circulation in the tropics. The ‘outbreak’ or ‘epidemic’ designation is commonly used in temperate regions to describe a more than fivefold increase in ILI activity, a criterion that could not be used in the tropics. Instead, year-round persistence with 9% lower activity during a 13-week period suggests that ILI transmission could experience a short low season in the tropics, inferred as late-February to mid-May in HCMC. Retooling mechanistic models to allow for or identify periods of low transmission may be the next step in understanding the long-term effects of this particular epidemiological driver. Identification of the driving forces of respiratory virus dynamics in the tropics is still very much an open question.

### Limitations

PCR-confirmed influenza reports around the Lunar New Year period were removed from the regression analysis because the laboratories were closed during this period. Data during this 2-week period each year were handled as missing data, despite higher human mobility and congregation patterns during this time that may have impacted respiratory virus transmission. This may lead to an unknown bias in the model inference, despite the fact that only 3% of all data points fall into these periods.

Difference in healthcare access between high-income and low-income settings may lead to differences in the populations being represented in syndromic surveillance reporting. For this reason, temperate and tropical ILI trends may not be directly comparable as general-population measures. Our study showed a high correlation between community surveillance and hospital-based surveillance as an internal validation (online supplemental figure S10), but comparison between Vietnam and Hong Kong or Singapore would be necessary to determine if healthcare access has any effect on regional patterns of respiratory disease transmission in Southeast Asia.^70 71^

Due to the nature of the data smoothing for the purpose of the analysis presented in this study, lag periods in the regression models were constrained to be multiples of 7 days for the models for ILI and 21 days for the models for ILI+. These were based on using 7-day smoothing to avoid reporting effects of weekends and 21-day test positivity rates. This limits the ability to include lag periods for climate factors of less than 7 days, which may represent important mechanisms in influenza transmission. The climate factors in the regression models carried low influence on the overall model fit, as shown by lower changes in AIC based on their inclusion compared with the inferred cycles.

The model fits shown from both the models for ILI and ILI+ in HCMC showed close fits to the data (figures 3C and 5C). However, the goodness-of-fit may be impacted by using the previously-fit step functions as covariates as these covariates were derived from the data. This potentially leads to overfitting in the models, where a function of the observed ILI(+) values was used as a predictor of ILI(+).

### Long-term outlook

The methods and results in this study can be extended into a forecasting framework in order to predict future peaks or incidence of influenza in HCMC. Similar work using statistical models to produce short-term forecasts of infectious disease burden has been applied to respiratory viruses as well as other non-respiratory human communicable diseases and vectorborne diseases.^72–77^ In the context of this study, the fitted regression models were shown to predict ILI and ILI+ with high accuracy (figures 3C and 5C), suggesting that extrapolating the models to predict future burden may also provide accurate forecasts. Extending the methods used in this study to produce a forecasting model is a natural extension of the current study. While regular forecasts of incidence may prove difficult due to the model’s setup to predict detrended data rather than actual incidence values, the forecasted trajectories would prove useful in identifying periods of relatively high ILI or ILI+ incidence.

If the approaches presented here lead to successful forecasts of ILI and ILI+ peaks, this may help inform prevention measures for influenza such as vaccination, public health messaging and preparation for increased incidence of hospitalisation. Influenza vaccination coverage in Vietnam is currently low,^78^ though efforts to increase vaccination among healthcare workers have been introduced in recent years. Noting the lack of strong annual seasons is important for designing vaccine campaigns in HCMC because there is little evidence to show that there is an optimal time for administering vaccines. Likewise, public health messaging and preparation in the public health and medical system have the potential to improve substantially if influenza and ILI patterns in Vietnam and other tropical regions can be better understood and forecast more accurately.

## Conclusions

Our study presents a unique pattern of the dynamics of influenza and other respiratory diseases in the tropical regions, represented by HCMC, Vietnam. Respiratory diseases showed both annual and nonannual cycles with 8.9% lower activity during spring. Influenza showed near-annual periodicities. By showing the different patterns between tropical regions and temperate regions, our study shows the importance of developing different prevention strategies in tropical regions than in temperate regions.

## Data Availability

Data are available in a public, open access repository. All the ILI and influenza data and code have been made publicly available at https://github.com/bonilab/tropicalflu-03FL-10years-communityILI-HCMC.
