# Supplementary material for: A combination of annual and nonannual forces drive respiratory disease in the tropics
Source: BMJ Glob Health. 2023 Nov 7;8(11):e013054. doi: 10.1136/bmjgh-2023-013054 (PMC10632872; doi:10.1136/bmjgh-2023-013054)
Supplement: Supplementary data [file bmjgh-2023-013054supp002.pdf]

Appendix S1 – Reflexivity Statement

1. How does this study address local research and policy priorities?

Infectious disease policy priorities in Vietnam center on dengue virus, tuberculosis, and HIV prevention. As malaria has nearly disappeared and HIV management is viewed as successful, Vietnam progressed during 1990-2010 to near middle-income status with disease priorities shifting to childhood vaccination coverage, outbreak response, and management of moderate and chronic diseases. In 2006, a national sentinel surveillance system was established for influenza. Ten years later, vaccination coverage for influenza was still low, but public awareness of and public health focus on influenza had shifted to make respiratory disease management an area of higher importance.

In August 2009, this study was initiated both (1) to improve the timeliness and scale of respiratory disease surveillance in Vietnam, and (2) to take advantage of 'big data' approaches that in the previous twelve months had made a big impression on the scientific community globally by demonstrating the power of large data sets to uncover relationships and influence policy.

The key to this Vietnam-led big data initiative was that we would not use social media posts or internet activity to generate high volume data streams, but rather we would establish a large network of community clinicians (in a city of ten million people) that were willing to report daily patient counts to a centralized reporting system. The major advantage of this approach was that each data point was traceable back to a particular doctor who had made a clinical assessment of a patient's respiratory symptoms.

2. How were local researchers involved in study design?

This study was based at the Oxford University Clinical Research Unit (OUCRU) in Ho Chi Minh City. Administratively, inside Vietnam, OUCRU is an NGO under the Ho Chi Minh City Department of Health and the Hospital for Tropical Diseases (HTD), with authority to conduct research with HTD given by the Ho Chi Minh City People's Committee. OUCRU hosts approximately 300 Vietnamese staff, scientists, and clinicians, and about 20 foreign researchers (including study PI MF Boni, from 2008-2017). All Vietnamese scientists listed as authors are currently or were once OUCRU-based scientists, including senior author Nguyen Van Vinh Chau who is currently head of the Hospital for Tropical Diseases and a strong supporter of this community-led effort.

The major study design effort took place during the pilot phase of the study (2009-2011, approximately) when the reporting approaches were discussed and modified, and when recruiting expanded to broaden the network of reporting clinicians. The major effort in recruitment, communication, and promotion came from Prof Tran Tinh Hien (senior clinician at HTD, and director of clinical research at OUCRU), and Dr Dong Thi Hoai Tam and Dr Ha Vinh (both senior clinicians at HTD, clinical researchers at OUCRU, and participating GPs in this study). Prof Tran Tinh Hien retired last decade and declined to be an author on this publication.

In addition to Prof Hien, Dr Hoai Tam, and Dr Ha Vinh, a group of about 5-10 participating clinicians regularly came to the workshops run for this study during 2009-2014. These doctors made suggestions, and allowed us to pilot several other reporting systems including log books and standard line-list approaches. These clinicians were also instrumental in designing the molecular diagnostic component of the study (launched May 2012) which ensured that no single clinic would have a high burden of enrolment and sample collection. This molecular surveillance design was accepted by

participating clinicians, allowed for further recruitment (through recommendations made by participating clinicians), and made this study component sustainable from 2012 to 2019.

3. How has funding been used to support the local research team?

Direct costs of 34,000 USD seed funds and 142,900 GBP of research funds from a Wellcome Trust Fellowship were used to fund this study from 2009 to mid-2017 (none of these funds went to the PI salary or any other salaries of non-Vietnamese researchers). In addition, 148,100 USD of supporting costs were sent from Pennsylvania State University to OUCRU for study operations from 2017 to 2019. This demonstrated that a surveillance system of this scale could be run for an annual local cost of 40,000 USD per annum, or 6 USD per ILI report – a helpful health-economic ratio that can be leveraged to put programs into place when foreign salaries do not need to be paid.

4. How are research staff who conducted data collection acknowledged?

The primary study coordinator Nguyen Thi Le Thanh is third author on the paper. The analysis lead from the previous mid-study publication (2018, *Influenza and Other Respiratory Viruses*, 12:742) Ha Minh Lam is fourth author on the paper; he advised on data assembly and data processing methods. All laboratory staff and clinical leads are authors on the paper. Dr Nguyen Van Vinh Chau promoted and helped expand the community-led effort and is a senior author on the paper.

Two study nurses Nguyen Thi Kim Cuong and Tran Thi Anh Tuyet are listed in the acknowledgements, per common authorship practice at OUCRU.

Some authorship positions were considered relative to authorship positions on the first 2018 paper from this study which had three equal-contribution lead authors (including first author Ha Minh Lam and then study coordinator Nguyen Thanh Hung) and two senior authors (Drs Dong Thi Hoai Tam and Ha Vinh, mentioned in the previous paragraphs).

5. Do all members of the research partnership have access to study data?

All ILI report data and aggregated molecular surveillance data are public and available to all study partners (the last portion of the time series will be made fully public upon publication). Patient data are available to Fuhan Yang, Nguyen Thi Le Thanh, Ha Minh Lam, Tran Thi Nhu Thao, Nguyen Ha Thao Vy, and Huynh Thi Phuong through the OUCRU CliRes database. Patient data would be available to Drs Ha Vinh and Dong Thi Hoai Tam, but these two clinicians did not participate in data analysis.

6. How was data used to develop analytical skills within the partnership?

This partnership lasted ten years and allowed several young scientists to develop analytical skills. Pre-doctoral research assistant Ha Minh Lam was mentored by Amy Wesolowski and Maciej Boni in initial data analysis, and this work formed the first publication in 2018. Doctoral researcher Fuhan Yang was mentored by Ha Minh Lam and NIH Postdoctoral Fellow Joseph Servadio and carried out

the second phase of the analysis. Pre-doctoral student Tran Dang Nguyen set up the original database in 2009 and 2010 and (mentored by Maciej Boni) developed the data skills needed to run a medium-sized mHealth study. Laboratory staff Tran Thi Nhu Thao and Huynh Thi Phuong are now pursuing their academic careers at Harvard University and the University of Münster.

7. How have research partners collaborated in interpreting study data?

The study's early workshops were used to interpret early-stage results and refine data presentation and visualization. The initial set of clinicians participating in these workshops were instrumental in evaluating the debate, inside the medical community in Vietnam, on whether respiratory disease incidence was believed to increase in one season or another. In addition, multi-year downward trends were observed in some reporting clinics, and study clinicians were indispensable in providing descriptions for why some of these trends existed and whether the declines corresponded to true reductions in respiratory pathogen circulation (they did not). Ha Minh Lam led the initial phase of results interpretation between 2014 and 2017, with mentorship from Amy Wesolowski and guidance from Dr Dong Thi Hoai Tam and Dr Ha Vinh.

8. How were research partners supported to develop writing skills?

Doctoral student Fuhan Yang and pre-doctoral student Ha Minh Lam took responsibility for drafting manuscripts for the two major manuscripts that have been based on this study. Mid-career academics on the study team supported and mentored students during the writing process.

9. How will research products be shared to address local needs?

All aggregate study data will be made public. Interim results from this study were shared with the wider medical community in Ho Chi Minh City and with the HCMC Department of Health through annual reports that were written through 2015. A public website – www.ili.vn – was created in June 2014 to provide daily real-time updates (like a weather app) on influenza-like illness activity in Ho Chi Minh City.

The next stages of respiratory disease management in Vietnam will need to undergo a prioritization process to separate out effort directed at SARS-CoV-2, influenza virus, and other respiratory pathogens. This effort will be led by author Pham Quang Thai at Vietnam's National Institutes of Hygiene and Epidemiology, supported by Marc Choisy at the Oxford University Clinical Research Unit.

10. How is the leadership, contribution and ownership of this work by LMIC researchers recognised within the authorship?

Authors NTLT and HML are the local coordination and analysis leads in Vietnam. HML was first author on the initial publication and fourth author on this publication for his mentorship role in helping the first author through the analysis. NVVC is a senior author on this paper (second-to-last).

10 out of 16 authors are from LMIC countries, and two of the remaining six authors spent the majority of the study period in an LMIC institution full-time during the study period.

11. **How have early career researchers across the partnership been included within the authorship team?**

At study initiation in 2009, PI MFB was an early career researcher who used this study as early data to apply for mid-career funding (Wellcome Trust Sir Henry Dale Fellowship) that would extend this community-led surveillance effort through to 2019. HML, JLS, FY are late-stage doctoral or early-stage postdoctoral scientists who have taken lead author positions on the two papers submitted from this study.

12. **How has gender balance been addressed within the authorship?**

Six authors are female and ten authors are male.

13. **How has the project contributed to training of LMIC researchers?**

The major training component, as listed above, has benefited HML and NTLT who have led the analysis and study management/coordination, respectively. TDN developed data management skills as a pre-doctoral student during 2009-2011. TTNT, NHTV, and HTP trained in molecular diagnostic analysis as part of the study team.

14. **How has the project contributed to improvements in local infrastructure?**

This project has not directly contributed to improvements in permanent infrastructure.

15. **What safeguarding procedures were used to protect local study participants and researchers?**

The scientific and public health leadership at OUCRU comes from Vietnamese researchers. The Scientific and Ethical Committee of the Hospital for Tropical Diseases has members who are Vietnamese clinicians and scientists (and no foreign members). Sufficient/adequate protection of study participants (privacy, injury, insurance, adverse effects) is evaluated by the HTD Scientific and Ethical Committee and monitored and implemented (if necessary) by the OUCRU Clinical Trials Unit. Protection of study participants is also evaluated by the Oxford Tropical Research Ethics Committee at the University of Oxford. Local researchers have agency and authority as study leads and hospital directors. Permission for foreign scientists to work in-country is determined by Vietnamese authorities.
