# Supplementary material for: A combination of annual and nonannual forces drive respiratory disease in the tropics
Source: BMJ Glob Health. 2023 Nov 7;8(11):e013054. doi: 10.1136/bmjgh-2023-013054 (PMC10632872; doi:10.1136/bmjgh-2023-013054)
Supplement: Supplementary data [file bmjgh-2023-013054supp001.pdf]

Supplementary Materials:

A combination of annual and nonannual forces drive respiratory disease in the tropics

Fuhan Yang[1], Joseph L Servadio[1], Nguyen Thi Le Thanh[2], Ha Minh Lam[2], Marc Choisy[2,3], Pham Quang Thai[4], Tran Thi Nhu Thao[2,5], Nguyen Ha Thao Vy[2], Huynh Thi Phuong[2], Tran Dang Nguyen[1,2], Dong Thi Hoai Tam[2], Ephraim M Hanks[6], Ha Vinh[2,7], Ottar N Bjornstad[1], Nguyen Van Vinh Chau[2,7], Maciej F Boni[1,2]

[1]Department of Biology and Center for Infectious Disease Dynamics, Pennsylvania State University, University Park, PA, 16802, United States

[2]Wellcome Trust Major Overseas Programme, Oxford University Clinical Research Unit, Ho Chi Minh City, Vietnam

[3]Centre for Tropical Medicine and Global Health, Nuffield Department of Medicine, University of Oxford, Oxford, UK

[4]National Institute of Hygiene and Epidemiology, Hanoi, Vietnam

[5]Department of Microbiology, Blavatnik Institute, Harvard Medical School, Boston, MA, 02115, United States

[6]Department of Statistics and Center for Infectious Disease Dynamics, Pennsylvania State University, University Park, PA, 16802, United States

[7]Hospital for Tropical Diseases, Ho Chi Minh City, Vietnam

S1 Text: Time series detrending

There are 7 clinics showing a long-term downtrend in %ILI. To remove the long-term trend and get a stationary time series, we detrended the %ILI by dividing each daily value by a 365-day moving average centered at that day for each clinic. We refer to this transformation as a ζ ("zeta")-score as it is related to an exponentiated z-score. This removes trends longer than 365 days and preserves the ratios between sequential daily reporting numbers. For influenza, we calculated daily ILI^+ as the product of the mean of the daily ILI ζ-score across all the clinics and the influenza positivity rate on that day to get a time series representing influenza incidence. Then we applied a 7-day moving average to smooth both the ILI ζ-score and ILI+ to filter out short-term noise. The 7-day moving smoothed ILI ζ-score and ILI+ are used for subsequent analysis.

S2 Text: ILI data from other surveillance systems

To compare ILI trends in HCMC with other regions, ILI data from the United States, four European countries (Belgium, France, Greece, Netherlands), Singapore, and Hong Kong were collected. The ILI data from the United States was downloaded from ILINet (US CDC https://www.cdc.gov/flu/weekly/index.htm). The weekly weighted %ILI from October 5, 1997 to December 29, 2019 from 10 HHS (Health and Human Services) regions were downloaded and then used as the overall ILI time series in the United States. The ILI data from Portugal, Belgium, Greece, and Netherlands were all downloaded from ECDC (https://flunewseurope.org/PrimaryCareData/SentinelVirologicalDetections). The weekly number of patients showing ILI symptoms per 100,000 population from October 5, 2015 to December 30, 2019 was used. The ILI data from France were downloaded from France Sentinelles network (https://www.sentiweb.fr/?lang=en). The weekly number of patients showing ILI symptoms per 100,000 population from November 3, 1984 to December 28, 2019 was used. The ILI data from Singapore were parsed from the Weekly Bulletin of the Singapore Ministry of Health (https://www.moh.gov.sg/resources-statistics/infectious-disease-statistics/2021/weekly-infectious-diseases-bulletin). The average daily number of patients seeking treatment for ILI per week was available from January 4, 2015 to December 28, 2019. ILI data from Hong Kong were downloaded from the Department of Health in Hong Kong

(https://www.chp.gov.hk/en/resources/29/304.html), from December 29, 2013 to December 28, 2019. All ILI data were converted into an ILI ζ-score time series with the same methods above.

S3 Text: Cyclic step function

We used simple step functions with k steps in a periodic cycle of length c. The function values and breakpoints were estimated by minimizing the Akaike Information Criterion (AIC) using normally distributed errors. We maximized a normal likelihood using the Nelder-Mead algorithm for step functions with cycle lengths, denoted c, ranging between 150 and 450 days (in increments of 5 days), and number of steps, denoted k, ranging from 2 through 8. Only the period that is longer than 15 days would be considered as one step in step function. The estimated parameters were selected from 100 optimized step functions with randomly selected initial parameters to ensure a global optimum was found. 95% confidence intervals were obtained via likelihood profiling.

For ILI+, the two-step function fit selected 330 days (AIC = -2414) and 385 days (AIC = -2405) as the dominant cycles in the overall ILI+ data, exhibiting a 105.4% (95% CI:[104.8 – 106.2]) increase during an 85-day high period (330-day inferred cycle) and a 106.2% (95% CI:[105.0 – 107.2]) increase during a 230-day high period (385-day inferred cycle, Fig. S6). The annual periodicity explains ILI+ less well (AIC = -2024) and shows weaker oscillation with a 55.1% (95% CI:[54.5 – 56.4]) increase from April 13 to July 18. The 8-step 330-day cycle (AIC = -2517) and 7-step 385-day cycle (AIC = -2491) were included in the final gamma-hurdle model.

S4 Text: Regression Covariates

We collected climate data from the NASA POWER project. Based on the criterion to only include climate factors that were reported to be biologically or epidemiologically associated with ILI transmission, we collected daily temperature, absolute humidity, and precipitation. We also included the 1-week, 2-week, 3-week lagged version of all climate variables because the effect of climate on ILI and influenza can be delayed. Absolute humidity was calculated using temperature and relative humidity:

$$AH = \frac{0.611 \times e^{\frac{17.502 \times T}{240.97+T}} \times 2.168 \times RH}{273.15+T} \quad \text{Eq.4}$$

Each climate predictor was normalized using z-score normalization with the mean and the standard deviation of the predictor time series and then smoothed using 7-day moving average. The school-term categorical variable is one from August 15 to June 1, when schools in HCMC are in session and zero otherwise.

S5 Text: Predictor importance

In multiple linear regression, the importance of each predictor in regression was measured as the averaged difference in R^2 when adding the predictor in all possible subsets of predictors in the model[33]. It is also referred as LMG in dominance analysis[34], defined as:

$$LMG(x_k) = \frac{1}{p}\sum_{i=0}^{p-1}\left(\sum_{\substack{S=\{x_1, x_2, \dots x_p | x_k\} \\ n(S)=i}} \frac{R^2(x_k \cup S) - R^2(S)}{\binom{p-1}{i}}\right) \quad \text{Eq.5}$$

where p is the number of predictors, S is all the subsets of predictors except x_k. In this way, we can calculate how much variance is explained by each predictor. We compared the predictor importance between the US ILI **ζ**-score and the ILI **ζ**-score in Ho Chi Minh City. For US ILI **ζ**-score, we started with fitting the step function given the cycle length is between 21 weeks and 64 weeks. The annual cycle was the fitted step function given the cycle length is 52 weeks, and the nonannual cycle was the best fitted step function out of the step functions when their cycle length is between 27 and 31 weeks (corresponding to the interval 190-220 days of the non-annual cycle in our ILI **ζ**-score). The 52-week cycle was always the best fitted step function in the ILI-**ζ** score in all the HHS regions. Next, we respectively regressed the ILI **ζ**-score in all the HHS regions using the same predictors as in the data in Ho Chi Minh City, including lagged climate factors from each HHS region, school term, 7-day lagged ILI **ζ**-score, and fitted cycles. We calculated the predictor importance from all the selected models using AIC-based selection, and we compared with the predictor importance of the model of ILI **ζ**-score in Ho Chi Minh City.

Supplementary Figures

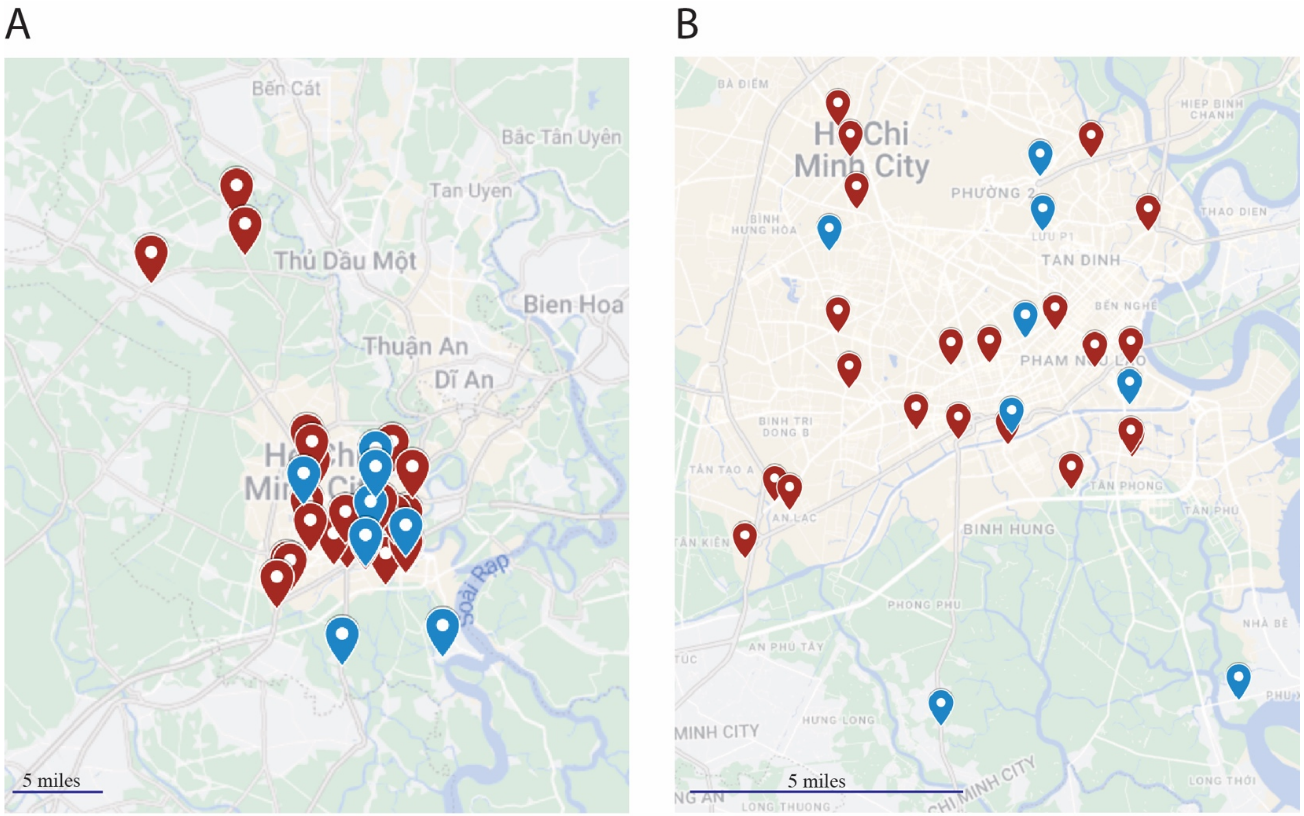

Figure S1. Geographical distribution of the 33 clinics selected for the analysis. 24 clinics participated in molecular influenza surveillance (red), and 8 clinics did not (blue). (**A**) Zoomed out map including three clinics in peri-urban Ho Chi Minh City. (**B**) Zoomed in map showing 29 clinics in the most populous central districts of Ho Chi Minh City. One clinic fell outside the boundaries of both maps.

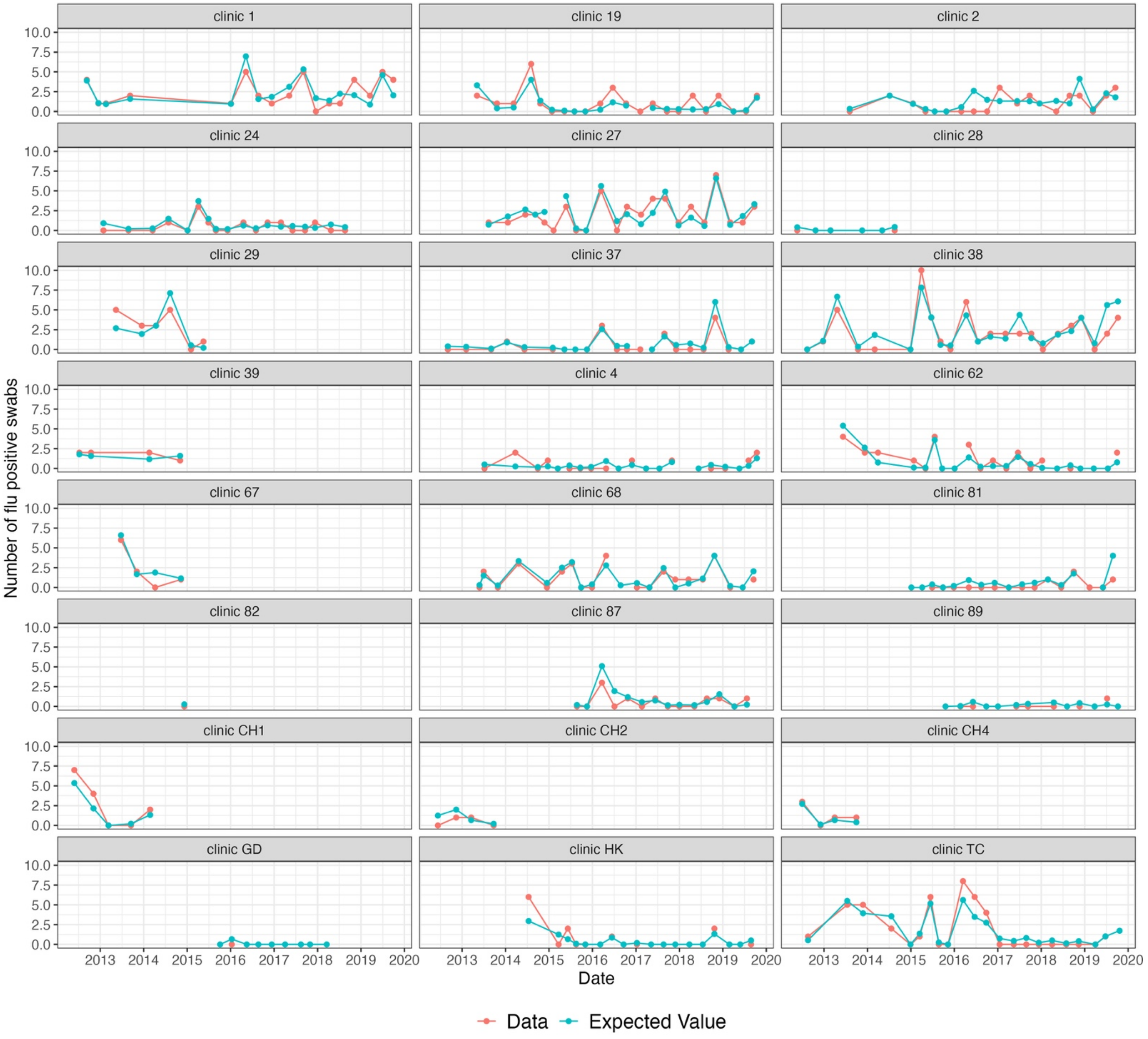

Figure S2. Validation of variation in ILI diagnosis using laboratory-confirmed influenza cases. We compare the number of influenza cases expected (blue) and the number of influenza cases reported (red) from 24 out of the selected 33 clinics. We used the influenza positive % in 21-day windows (among swabbed ILI patients, across all clinics) as a baseline, and calculated the number of expected influenza cases (blue) for each clinic using this baseline percentage. With the possible exception of clinic 19, the clinics did not show consistently higher or lower trends than their expected values, suggesting that there is no bias in ILI diagnosis.

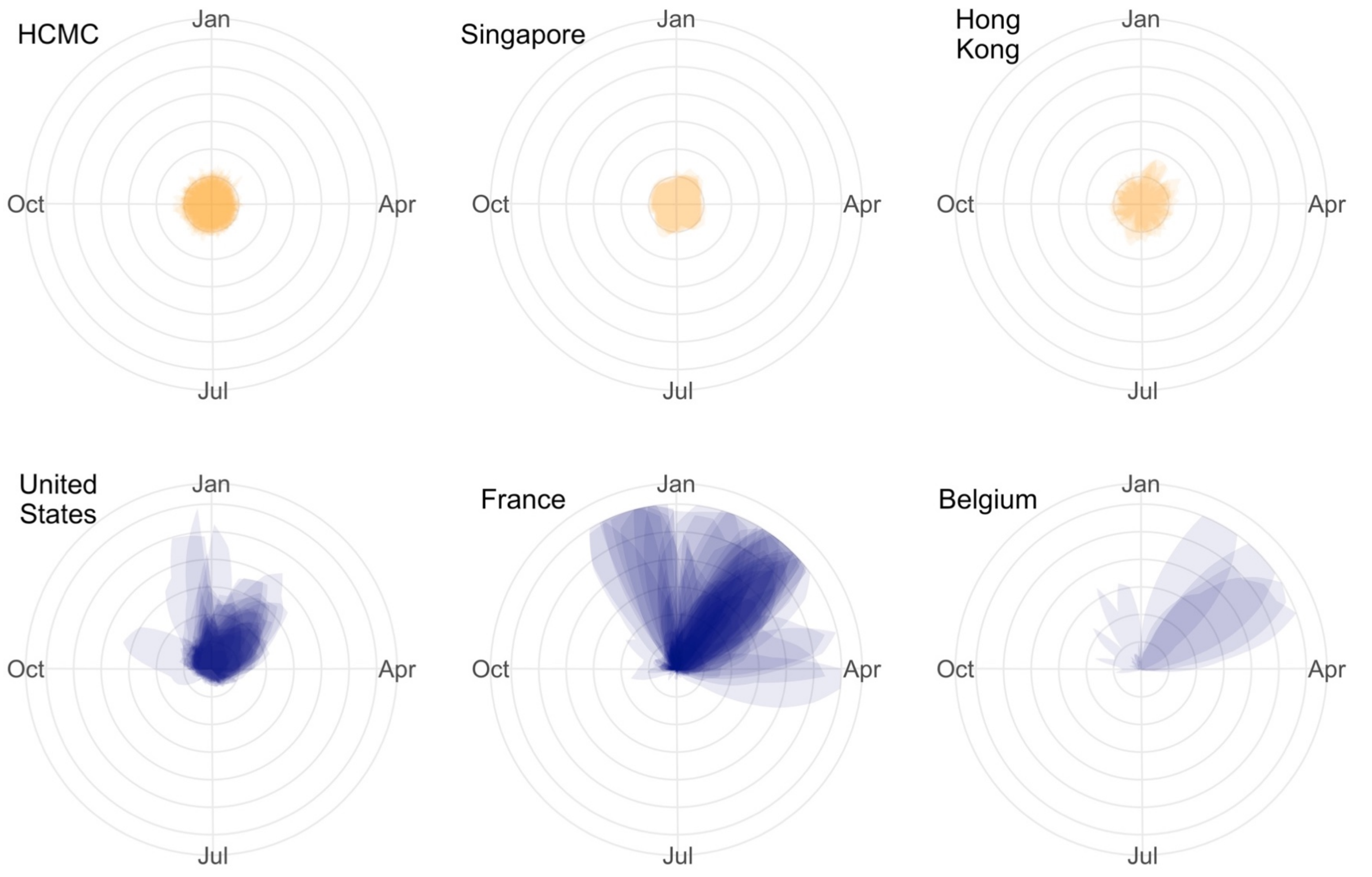

Figure S3. Circular plots of weekly ILI ζ -score from HCMC, Singapore, Hong Kong, the United States, France, and Belgium (data sources: S2 Text). The orange color indicates tropical or subtropical regions, the blue color indicates temperate regions. The data from each year was visualized in a circular time scale. The range of each plot is [0,6]. The weekly ILI ζ -score from HCMC is calculated by averaging the daily ILI ζ -score within one week.

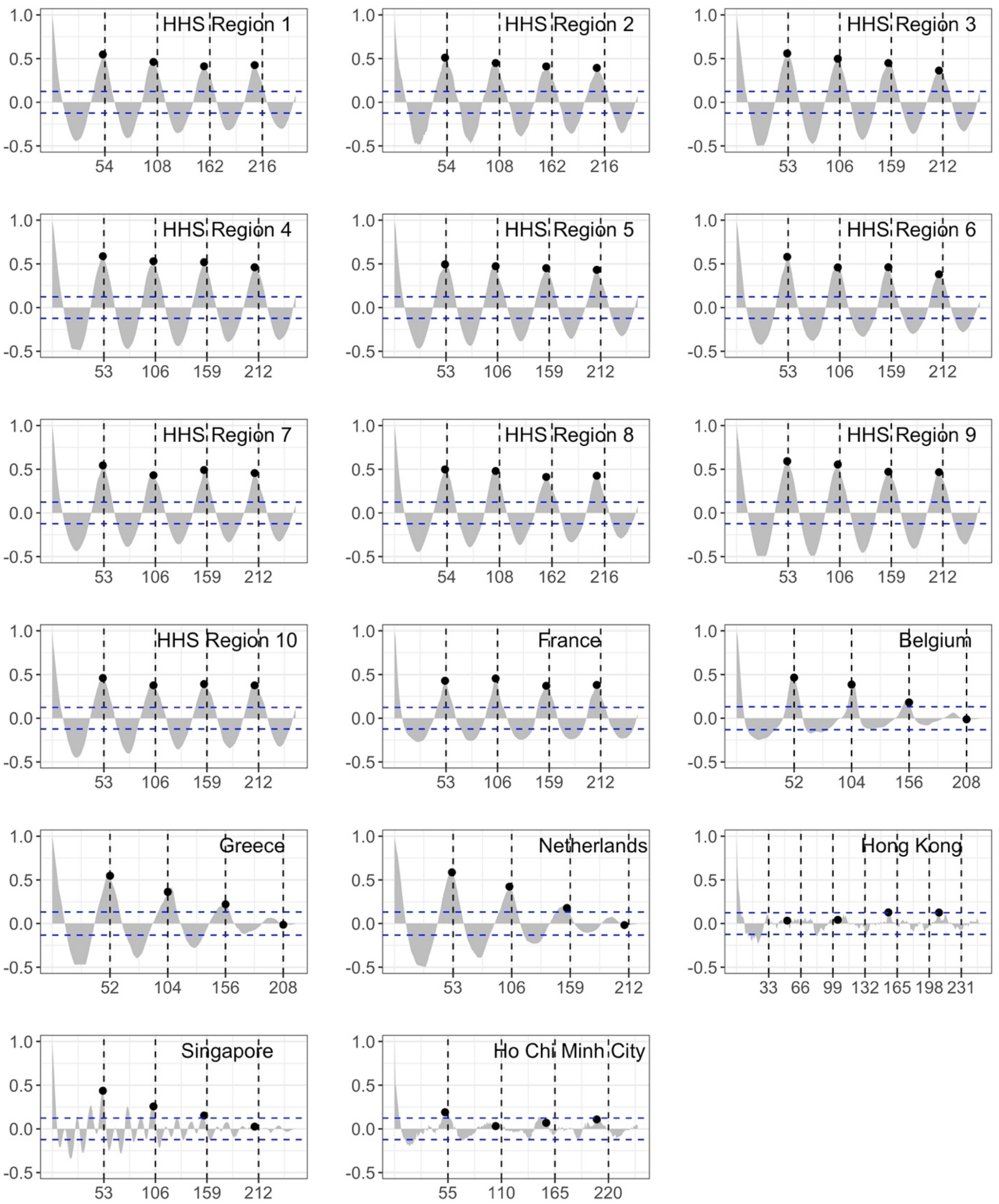

Figure S4. ACF plots of the weekly ILI ζ -score from locations in temperate regions and tropical regions (data sources: S2 Text). Compared to temperate regions, the ILI and in HCMC exhibit weak annual seasonality. Weak seasonality is also observed in Hong Kong and Singapore.

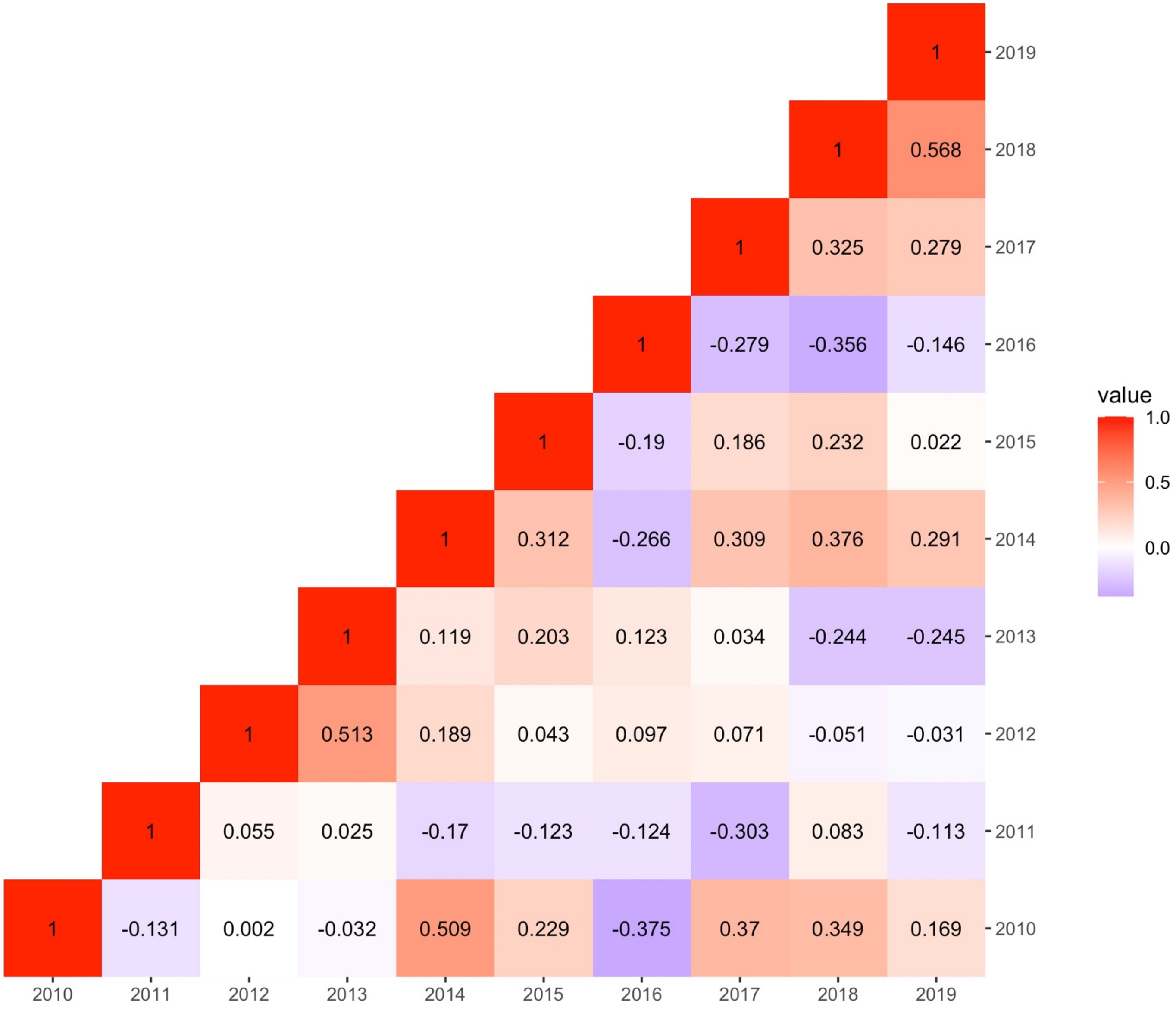

Figure S5. Pearson's correlation of ILI ζ -score between different years from 2010 to 2019. The highest observed correlation is seen between 2018 and 2019.

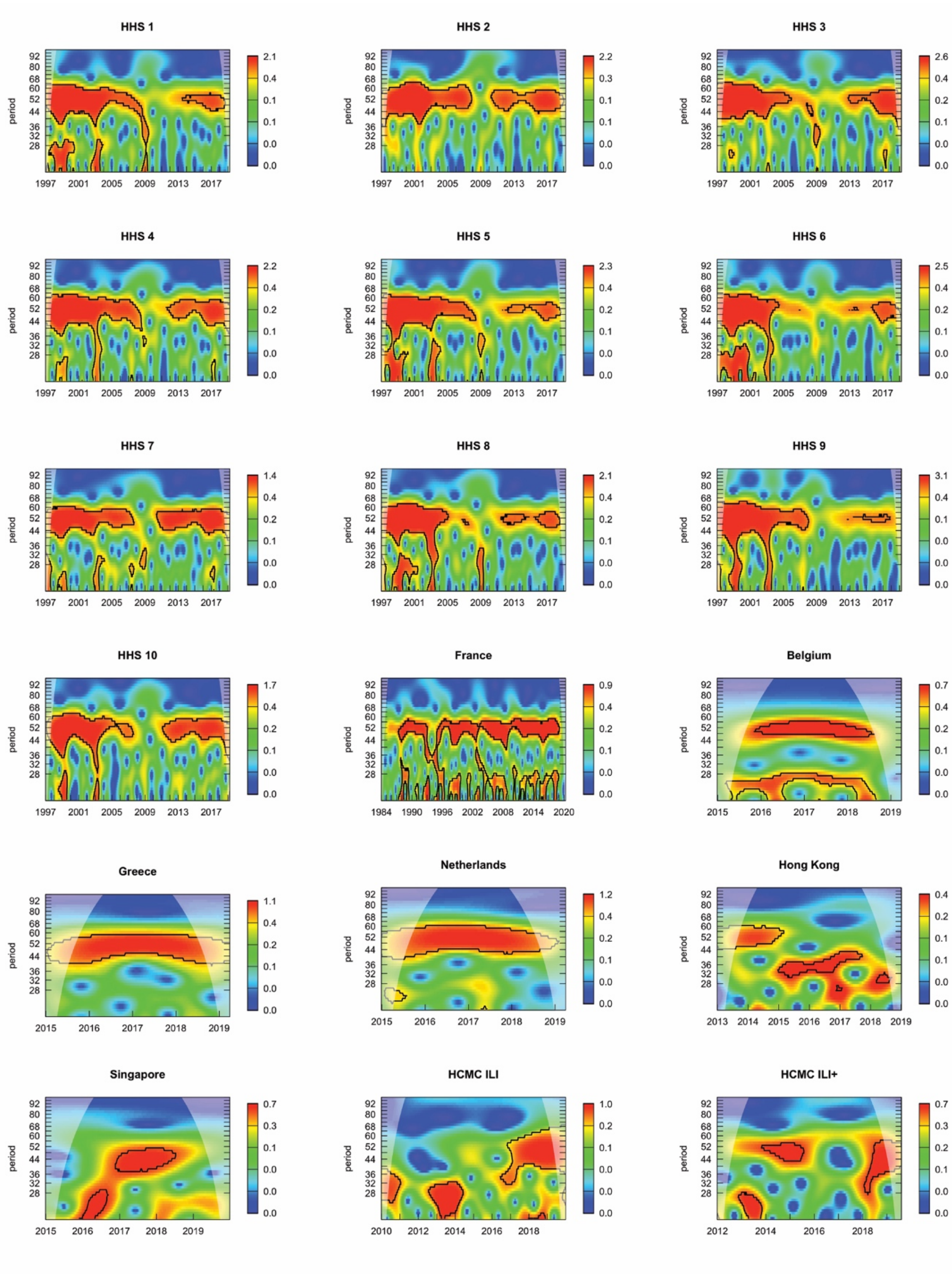

Figure S6. Wavelet transform of the ILI ζ -score in ten HHS regions from the United States, four European countries (France, Belgium, Greece, and Netherlands), subtropical city Hong Kong, and tropical country Singapore, along with ILI+ from HCMC. Continuous annual seasonality (52 weeks) is only observed in temperate regions. The disruption of annual seasonality in the HHS regions can be seen during the 2009 H1N1 pandemic.

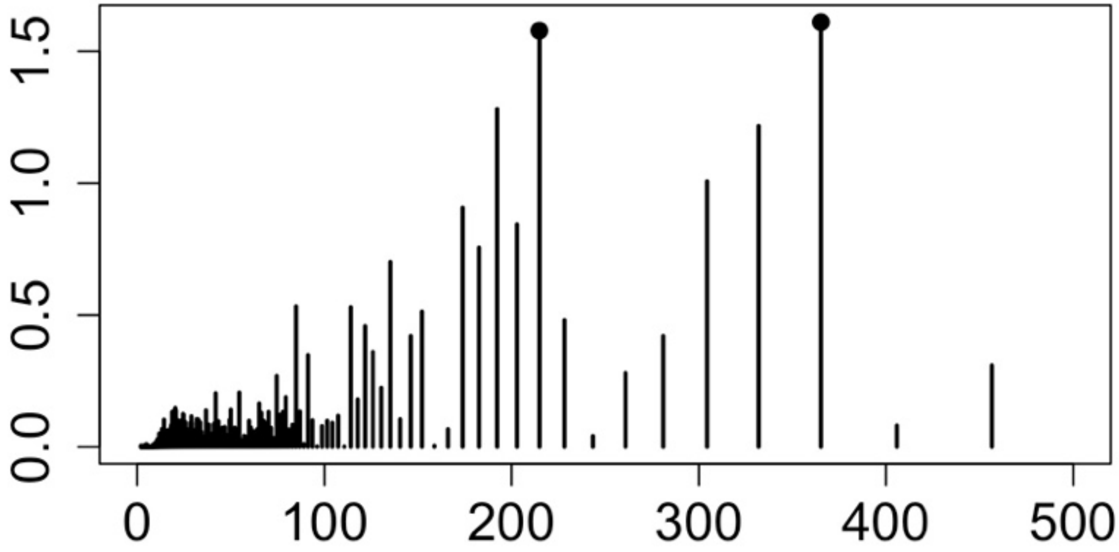

Figure S7. Discrete Fourier transform of ILI ζ -score. The signal of 215-day cycle and 365-day cycle are equivalently strong.

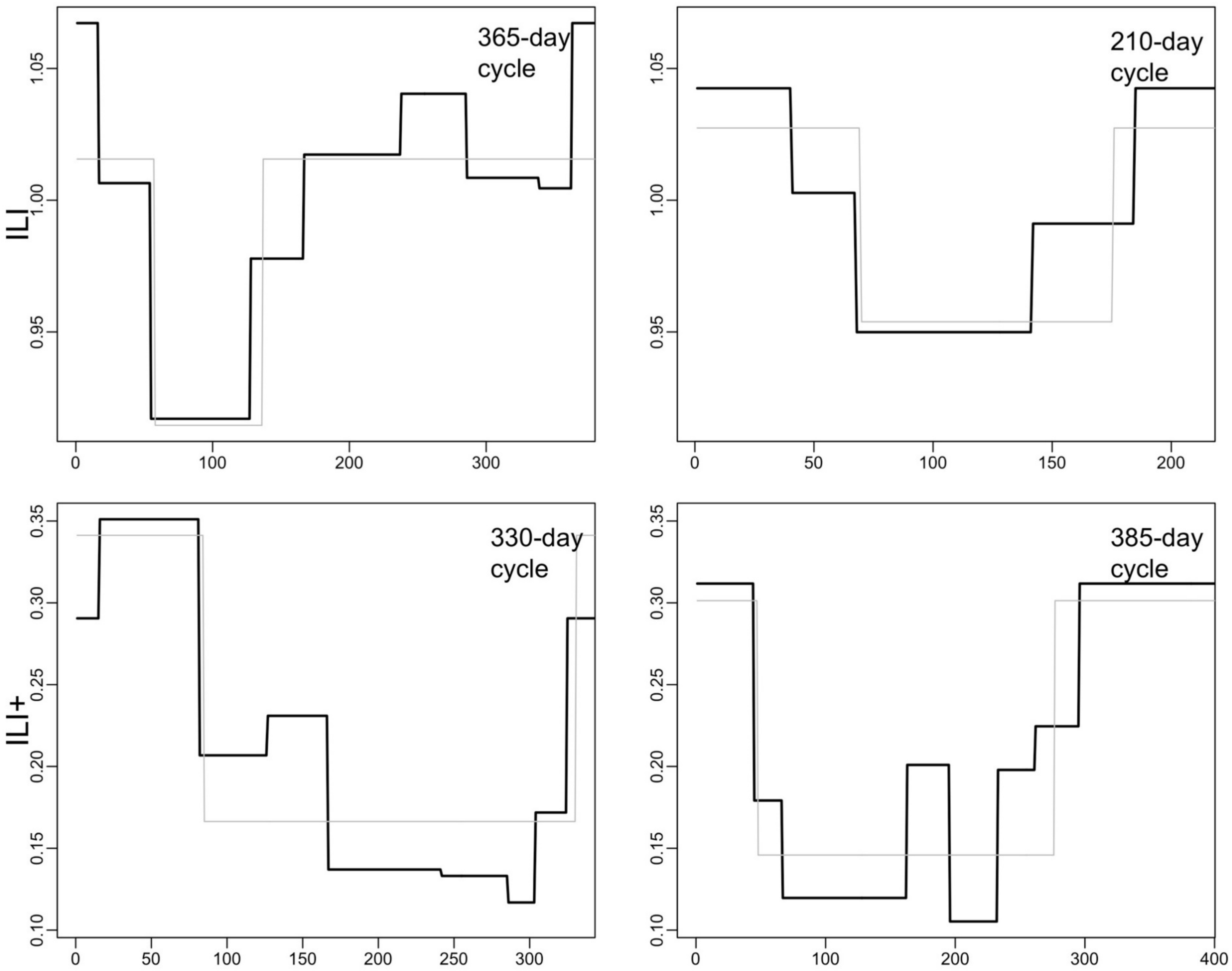

Figure S8. Inferred step function cycles of ILI ζ-score and ILI+. The grey lines show the inferred 2-step cycles. The black lines show the multi-step cycles with lowest AIC.

**Coefficients**	**Estimate**	**95% Confidence interval**	**ΔAIC**
**Lognormal Model of ILI zeta score**			
**Intercept**	-1.193***	[-1.348,-1.038]	NA
**7-day lagged ILI zeta score**	0.386***	[0.357,0.416]	611
**Non-annual cycle**	0.652***	[0.559,0.745]	183
**Annual cycle**	0.538***	[0.422,0.655]	79
**Absolute humidity**	-0.02***	[-0.028,-0.013]	26
**14-day lagged absolute humidity**	0.019***	[0.01,0.028]	14
**7-day lagged precipitation**	0.013***	[0.006,0.02]	11
**21-day lagged precipitation**	-0.009**	[-0.016,-0.002]	4
**21-day lagged temperature**	0.006*	[0001,0.01]	3
**21-day lagged absolute humidity**	0.007	[-0.002,0.016]	0.6
* p < 0.05<br>** p < 0.01<br>*** p < 0.001			

Table S1. Regression coefficients from the lognormal model of ILI zeta score. The weather predictors were scaled using z-score normalization.

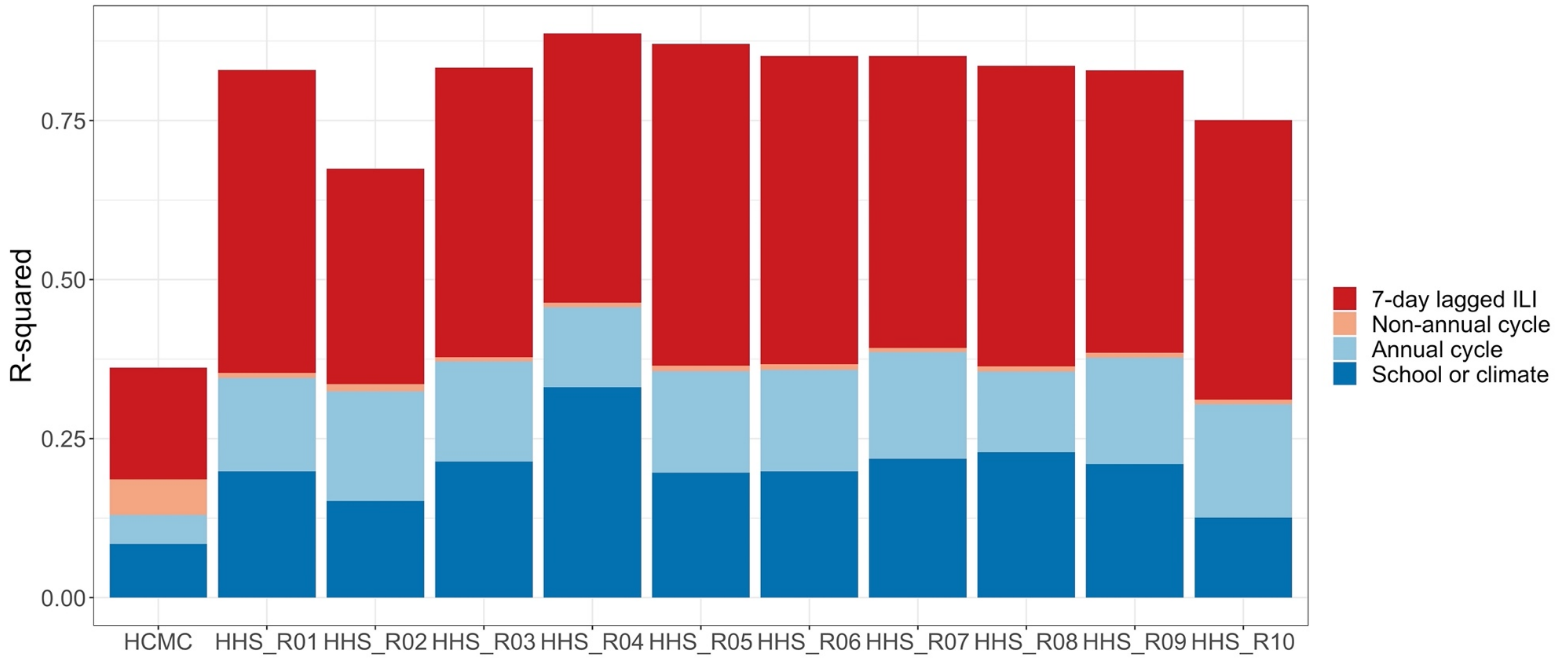

Figure S9. R^2 partitioned to the predictors with school term and climatic factors combined from the models of the ILI ζ -score from HCMC and ten HHS regions from the United States.

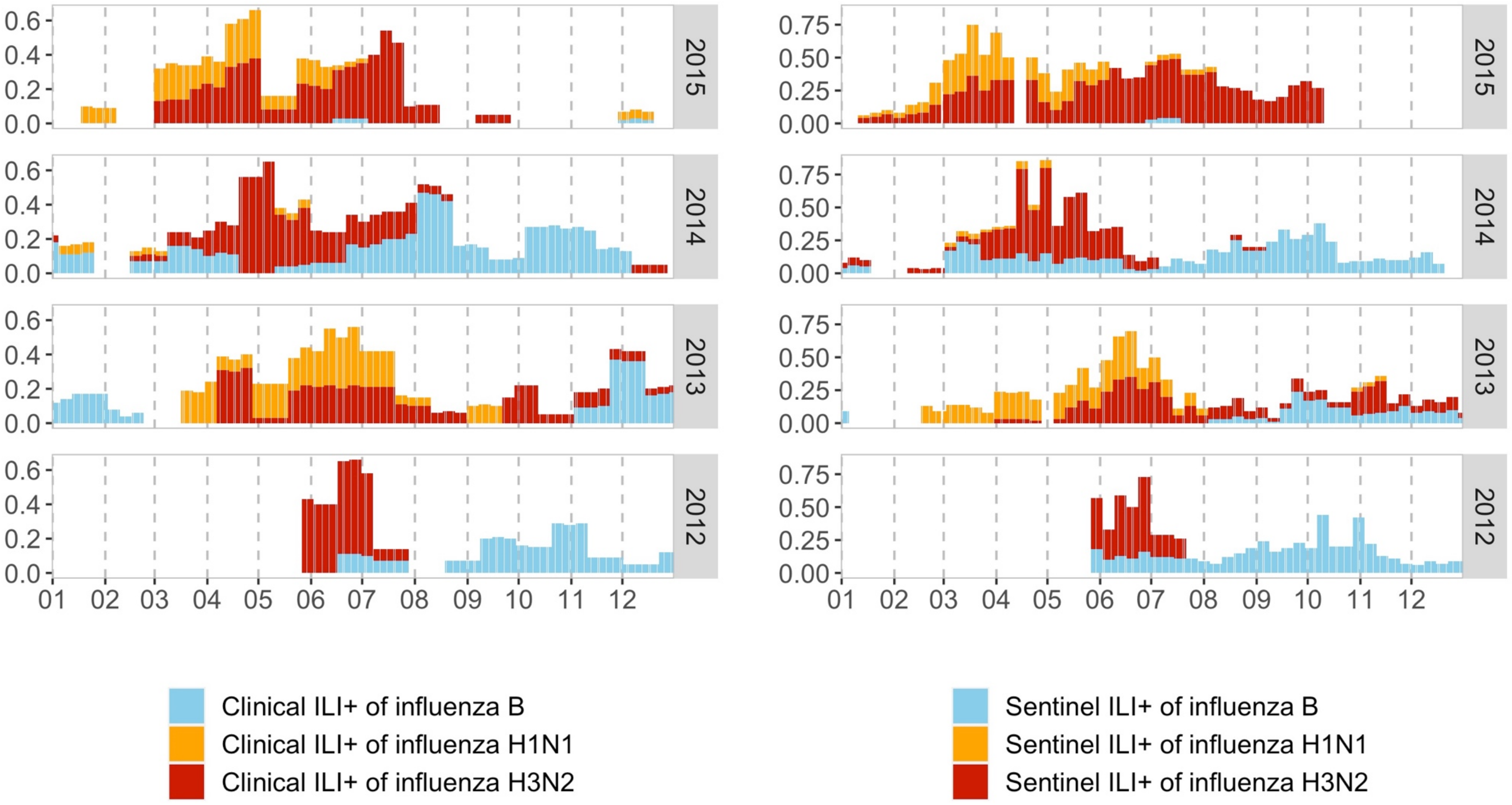

Figure S10. ILI+ from HCMC surveillance system (Left) and Hospital for Tropical Diseases in Ho Chi Min City (Right). Weekly ILI+ is calculated to be comparable to the hospital's weekly ILI+. During the period when two time series are both available, ILI+ shows similar trends in both settings.

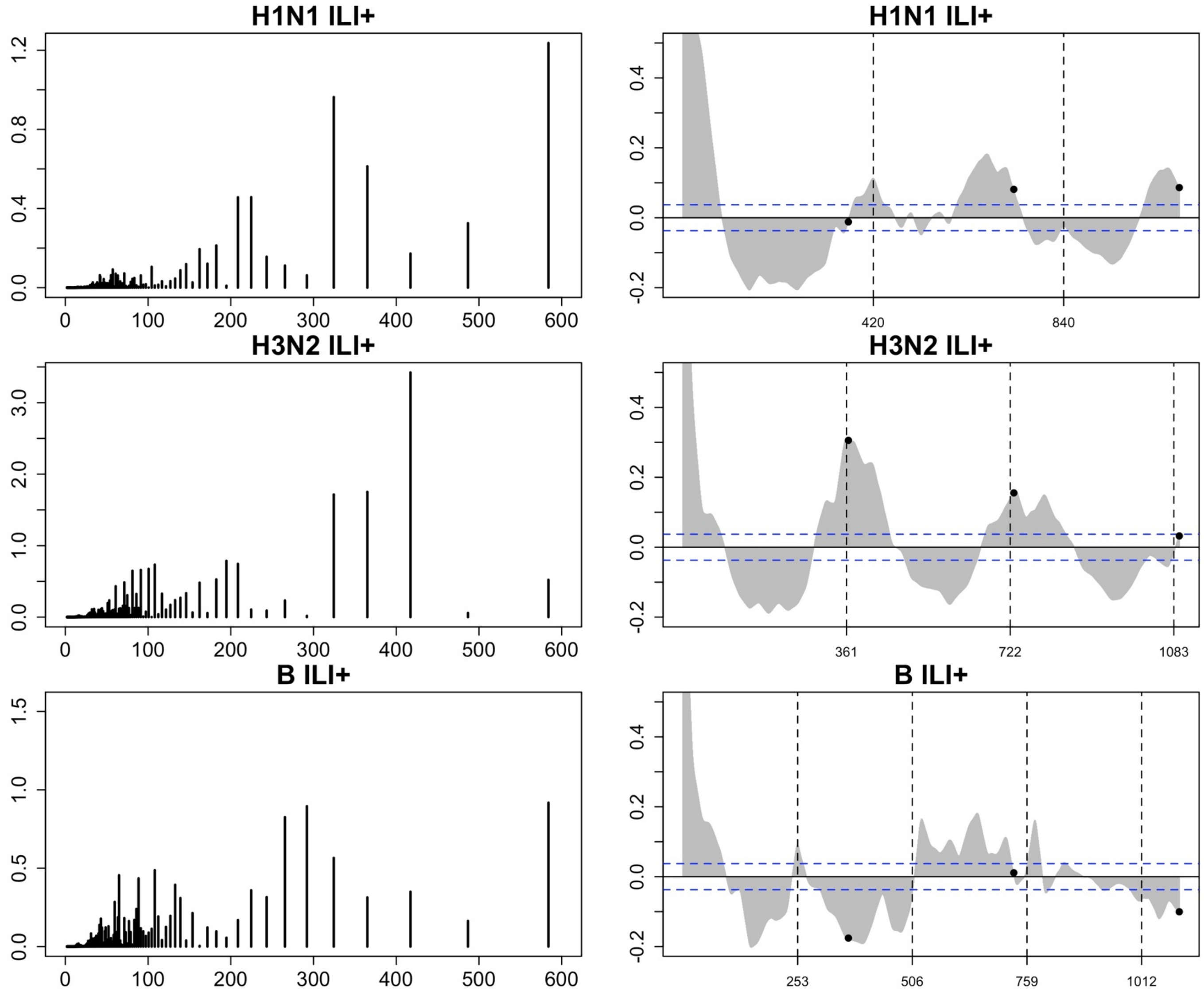

Figure S11. Discrete Fourier transform and ACF plots of subtype ILI+. Based on the spectral density and the ACF coefficients, H3N2 ILI+ showed the strongest annual periodic signal, while H1N1 ILI+ and influenza B ILI+ showed irregular pattern in the absence of annual seasonality.

**Coefficients**	**Estimate**	**95% Confidence Interval**	**ΔAIC**
**Logistic Model of ILI$^+$**			
**Intercept**	10.687***	[6.143, 15.23]	NA
**21-day lagged ILI+**	10.114***	[8.45, 11.778]	242
**Temperature**	-0.471***	[0.355, 0.587]	70
**21-day lagged rainfall**	-0.108***	[0.067, 0.149]	22
**330-day cycle**	18.888***	[10.208, 27.567]	17
**School term**	0.911***	[0.414, 1.408]	11
**14-day lagged absolute humidity**	-1.679**	[-2.716, -0.643]	9
**385-day cycle**	8.706**	[2.141, 15.271]	5
**Rainfall**	0.061*	[0.006, 0.115]	3
**7-day lagged rainfall**	0.058*	[0.006, 0.11]	3
**Gamma Model of ILI$^+$ (link = 'log')**			
**Intercept**	-4.087***	[-4.801, -3.373]	NA
**21-day lagged ILI+**	1.476***	[1.317, 1.635]	326
**330-day cycle**	-5.659***	[-6.726, -4.592]	103
**21-day lagged temperature**	0.078***	[0.061, 0.095]	81
**385-day cycle**	4.111***	[2.997, 5.226]	49
**21-day lagged rainfall**	0.013***	[0.006, 0.208]	10
**7-day lagged rainfall**	0.011**	[0.004, 0.018]	7
**Rainfall**	0.011**	[0.003, 0.176]	7
**7-day lagged absolute humidity**	0.362**	[0.116, 0.607]	6
**School term**	0.104**	[0.032, 0.177]	6
**21-day lagged absolute humidity**	-0.297*	[0.006, 0.021]	5
*** p < 0.05**<br>**** p < 0.01**<br>***** p < 0.001**			

Table S2. The coefficients from the gamma-hurdle model of ILI+. The weather predictors were scaled using z-score normalization.
